# Cell nonautonomous roles of NHR‐49 in promoting longevity and innate immunity

**DOI:** 10.1111/acel.13413

**Published:** 2021-06-22

**Authors:** Nikki Naim, Francis R. G. Amrit, Ramesh Ratnappan, Nicholas DelBuono, Julia A. Loose, Arjumand Ghazi

**Affiliations:** ^1^ Department of Pediatrics University of Pittsburgh School of Medicine Pittsburgh PA USA; ^2^ Departments of Developmental Biology and Cell Biology and Physiology University of Pittsburgh School of Medicine Pittsburgh PA USA

**Keywords:** ageing, *C. elegans*, cell non‐autonomous, health span, immunity, longevity, NHR‐49, stress response

## Abstract

Aging and immunity are inextricably linked and many genes that extend life span also enhance immunoresistance. However, it remains unclear whether longevity‐enhancing factors modulate immunity and longevity by discrete or shared mechanisms. Here, we demonstrate that the *Caenorhabditis elegans* pro‐longevity factor, NHR‐49, also promotes resistance against *Pseudomonas aeruginosa* but modulates immunity and longevity distinctly. NHR‐49 expression increases upon germline ablation, an intervention that extends life span, but was lowered by *Pseudomonas* infection. The immunosusceptibility induced by *nhr*‐*49* loss of function was rescued by neuronal NHR‐49 alone, whereas the longevity diminution was rescued by expression in multiple somatic tissues. The well‐established NHR‐49 target genes, *acs*‐*2* and *fmo*‐*2*, were also differentially regulated following germline elimination or *Pseudomonas* exposure. Interestingly, neither gene conferred immunity toward Gram‐negative *Pseudomonas*, unlike their known functions against gram‐positive pathogens. Instead, genes encoding antimicrobial factors and xenobiotic‐response proteins upregulated by NHR‐49 contributed to resistance against *Pseudomonas*. Thus, NHR‐49 is differentially regulated by interventions that bring about long‐term changes (life span extension) versus short‐term stress (pathogen exposure) and in response it orchestrates discrete outputs, including pathogen‐specific transcriptional programs.

## Graphical abstract

Longevity and immune health are intimately connected, but it is unclear whether the two can be genetically uncoupled. This study shows that a worm transcription factor, NHR‐49, promotes both longevity and immunity distinctly. NHR‐49 expression in neurons is critical for immunoresistance, whereas its presence in multiple tissues can improve longevity. It controls the production of antibacterial proteins to combat infection and alters fat breakdown to increase life span.

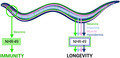

## 1 INTRODUCTION

Recent advances in aging research have led to a shift in focus from increasing life span to improving health span, a concept that encompasses measures of physiological health, including stress resistance (Keith et al., 2014; Melov, 2016; Richardson et al., 2016; Sierra, 2016). A strong positive correlation exists between longevity and stress resistance in model organisms and in nature, and many genes that increase life span have been reported to enhance stress resilience (Epel & Lithgow, 2014; Kirkwood et al., 2000; Murakami, 2006; Zhou et al., 2011). However, descriptions of long‐lived mutants that do not exhibit elevated stress resistance, and *vice versa*, are reported in literature along with instances of pro‐longevity genes that do not alter stress resistance (Arum & Johnson, 2007; Dues et al., 2017; Meissner et al., 2004). Recently, we and others have identified genes that promote longevity but repress stress resilience demonstrating that these attributes are physiologically distinct (Amrit et al., 2019; Kew et al., 2020). Nonetheless, a large fraction of known pro‐longevity genes promote stress resistance (Zhou et al., 2011), but whether they govern these processes by shared or distinct mechanisms remains largely unknown.

Immunity is central to an organism’s stress response and thus an integral measure of health span (Keith et al., 2014; Richardson et al., 2016). Age‐related increase in disease susceptibility occurs across species, including in humans (Nikolich‐Zugich, 2018), as demonstrated by the COVID‐19 pandemic’s disproportionate impact on the elderly (Cunha et al., 2020). While both the adaptive and innate immune systems undergo age‐associated changes, the latter is a major focus of contemporary aging biology as inflammaging, a derailment of innate immunity causing chronic inflammation, has been postulated to underlie age‐related pathologies (Franceschi & Campisi, 2014; Fulop et al., 2018, 2019). Moreover, longevity‐promoting genes and drugs such as FOXO3A and Metformin, respectively, have been shown to ameliorate the inflammaging profile of older immune cells toward that of a younger cohort (Bharath et al., 2020; Leavy, 2014; Lee et al., 2013). To what extent such genes and drugs modulate immune status directly is unclear and has topical relevance to human aging biology.

Studies in the nematode, *Caenorhabditis elegans*, have been instrumental in identifying fundamental aging mechanisms, including the discovery that signals from the reproductive system influence life span and stress resistance (Amrit & Ghazi, 2017a). In worms, removal of a population of totipotent germline‐stem cells (GSCs) increases life span dramatically (Hsin & Kenyon, 1999). GSC‐less animals also display extraordinary metabolic adaptability and resilience against stressors such as heat, DNA damage or infections by gram‐positive and gram‐negative pathogens (Alper et al., 2010; Amrit & Ghazi, 2017a). The increased longevity of GSC‐less worms is attributable to a network of transcription factors activated in somatic cells, including DAF‐16, worm homolog of FOXO3A (Amrit & Ghazi, 2017a; Ziv & Hu, 2011). Similar phenomena observed in other species such as flies and mice (Benedusi et al., 2015; Cargill et al., 2003; Flatt et al., 2008; Mason et al., 2009) as well as human population studies (Levine et al., 2016; Min et al., 2012) suggest that the reproductive control of aging may be widespread in nature and involve conserved genetic mechanisms (Lunetta et al., 2007; Pelosi et al., 2013; Vilchez et al., 2012). Previously, we identified a group of nuclear hormone receptors (NHRs) critical for GSC‐less longevity, including NHR‐49 (Ratnappan et al., 2014), worm functional homolog of the vertebrate energy/metabolism regulator, and peroxisome proliferator‐activated receptor alpha (PPARα). We showed that NHR‐49 coordinately upregulates the expression of genes involved in fatty acid β‐oxidation as well as lipid desaturation and elongation to preserve lipid homeostasis and promote longevity (Ratnappan et al., 2014). NHR‐49 also orchestrates the transcriptional responses to acute starvation and oxidative stress (Goh et al., 2018; Van Gilst, Hadjivassiliou, & Yamamoto, 2005). But, whether NHR‐49 promotes pathogen resistance in GSC‐less worms is unknown.

In worms and other organisms, many cell nonautonomous regulators of longevity and stress resistance, including immunoresistance, have been identified (Hoffman & Aballay, 2019; Medkour et al., 2016; Morimoto, 2020; Wani, 2020). For instance, DAF‐16/FOXO3A is sufficient in intestinal cells to confer longevity on GSC‐less worms (Libina et al., 2003) and *Drosophila* dFOXO in the fat body increases life span (Hwangbo et al., 2004). But, intestinal DAF‐16 cannot rescue the heat resistance of GSC‐less animals and provides little life span benefit to mutants of the insulin/IGF1 receptor, *daf*‐*2*, whose longevity is also completely reliant upon it (Kenyon et al., 1993; Libina et al., 2003). Thus, site‐of‐action and physiological context are both critical in determining gene function (Amelio & Melino, 2020; Ben‐David & Amon, 2020; Uschner & Klipp, 2019). Neuronal NHR‐49 promotes longevity mediated by AMP‐activated protein kinase, AMPK (Burkewitz et al., 2015). But, where the protein acts to modulate life span in GSC‐less animals, or the stress response pathways it controls, remains unstudied.

In this study, we describe a role for NHR‐49 in the innate immune response against the gram‐negative pathogen *Pseudomonas aeruginosa* in long‐lived, GSC‐less animals as well as normal, fertile adults. We demonstrate that NHR‐49 is differentially influenced by GSC loss versus pathogen exposure. While NHR‐49 expression in any somatic tissue rescued longevity, only neuron‐derived protein could promote pathogen resistance in multiple genetic backgrounds. These distinct regulatory effects also extended to the expression of well‐established NHR‐49 target genes, *acs*‐*2*, and *fmo*‐*2*, neither of which were required to defend against *P*. *aeruginosa*, unlike their known roles against other pathogens (Dasgupta et al., 2020; Wani et al., 2021). Instead, NHR‐49 targets encoding antimicrobial factors and xenobiotic‐response proteins contributed toward *Pseudomonas* resistance. Overall, our data suggest that NHR‐49 directs distinct responses to short‐term stimuli such as pathogen attack versus long‐term life span changes and orchestrates pathogen‐specific transcriptional programs.

## 2 RESULTS

### 2.1 NHR‐49 co‐regulates with DAF‐16 and TCER‐1 the expression of genes essential for germline‐less longevity

**FIGURE 1:**
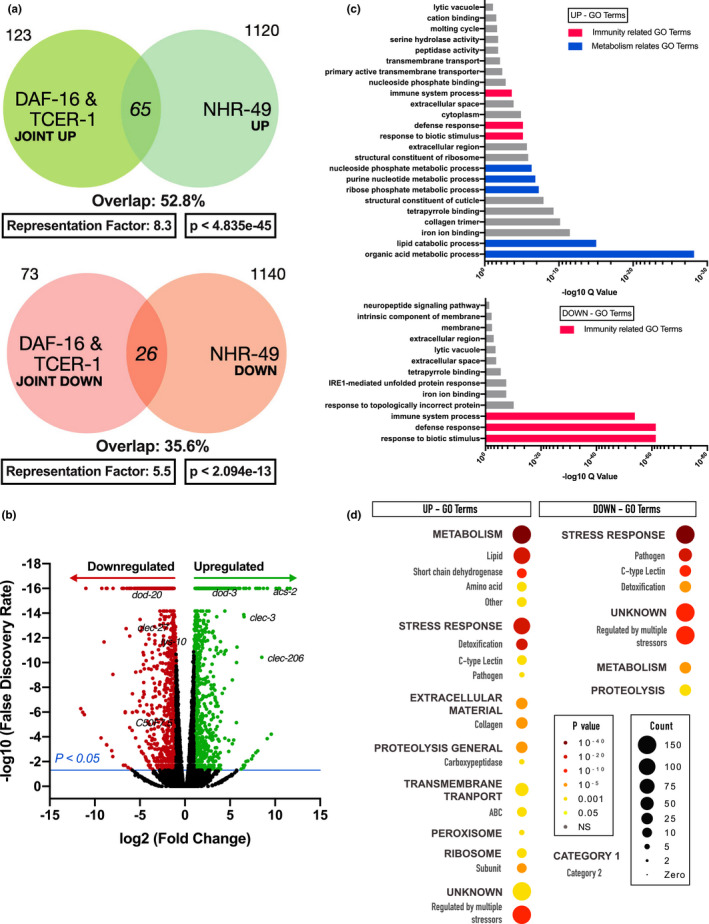
NHR‐49 dictates a transcriptome upon germline loss that is enriched for innate immunity genes. (a) Venn diagram comparing the genes upregulated (top, 1,120) and downregulated (bottom, 1,140) by NHR‐49 in *glp*‐*1* mutants with genes jointly upregulated (top, 188) and downregulated (bottom, 99) by DAF‐16 and TCER‐1 following germline loss (Amrit et al., 2016). (b) Volcano plot of gene expression changes between *glp*‐*1* and *nhr*‐*49*;*glp*‐*1* animals. Differentially expressed genes highlighted as red (NHR‐49 DOWN) and green (NHR‐49, UP). Absolute log fold change >2, *p* value <0.05 and FDR of <2. Immune‐gene classes and factors discussed in this study are labeled. (c) Wormbase Gene Set Enrichment Analysis (GSEA) of NHR‐49 targets identified metabolism (blue) and stress response (red) categories as being enriched. (d) Gene ontology (GO) term analysis using WormCat, a *C*. *elegans* identified pathogen response as one of the most enriched terms in both the UP class and DOWN Class

Previously, we demonstrated that *nhr*‐*49* inactivation abrogates the enhanced life span of temperature sensitive, sterile *glp*‐*1* mutants (Arantes‐Oliveira et al., 2003), a well‐established model for GSC‐less longevity (Amrit & Ghazi, 2017a). To identify the transcriptional changes orchestrated by NHR‐49 upon GSC removal, we compared the transcriptomes of *glp*‐*1* versus *nhr*‐*49*;*glp*‐*1* mutants using RNA‐Seq. We found that NHR‐49 controlled the transcriptional upregulation of 1,120 genes (UP class) and downregulation of 1,140 genes (DOWN class) in *glp*‐*1* mutants (Figure S1a, Table S1a,b). Since our previous studies had shown that *nhr*‐*49* is transcriptionally upregulated upon GSC loss by the joint action of two transcription factors, DAF‐16 and TCER‐1 (Ratnappan et al., 2014), we examined the overlap between NHR‐49 UP and DOWN targets with genes whose expression is altered by DAF‐16 and/or TCER‐1 in *glp*‐*1* mutants (Amrit et al., 2016). Strikingly, 53% of genes upregulated in *glp*‐*1* mutants *jointly* by DAF‐16 and TCER‐1 (JOINT UP) (Amrit et al., 2016) were also identified within the NHR‐49 UP class (65/123, R factor 8.3, *p* < 4.853e‐45) (Figure 1a). The overlap with genes specifically upregulated by either of these proteins was also significant ranging from ~24% (DAF‐16 UP) to ~40% (TCER‐1 UP) (Figure S1b). The NHR‐49 DOWN class also showed a much higher overlap with the genes jointly downregulated by DAF‐16 and TCER‐1 (JOINT DOWN) (~36%, 26/73, R 5.5, *p* < 2.094e‐13) (Figure 1a) as compared to genes specifically downregulated by either factor alone (Figure S1b). Notably, 35 of these NHR‐49 UP genes were identified in our previous studies as being essential for *glp*‐*1* mutants’ longevity (Figure S1c) (Amrit et al., 2016; Ratnappan et al., 2014). Hence, our RNA‐Seq analysis identified NHR‐49‐dependent, functionally relevant genes essential for the life span extension induced by GSC loss.

### 2.2 NHR‐49 widely modulates the expression of innate immunity genes

Based on NHR‐49’s known roles in regulating fat metabolism (Goh et al., 2018; Svensk et al., 2013; Van Gilst, Hadjivassiliou, Jolly, et al., 2005), we anticipated its targets to be enriched for lipid metabolic functions. Gene Ontology (GO) analysis of the RNA‐Seq data (Amrit & Ghazi, 2017b) revealed that, while metabolic functions were indeed highly represented among NHR‐49 targets, some of the most enriched GO terms related to stress response, especially immune response (Figure 1b,c). Analysis of this data through WormCat, a *C*. *elegans* bioinformatics platform that allows greater refinement of functional categories within enriched groups (Holdorf et al., 2020), substantiated these observations. Within the UP class, stress response, particularly pathogen response, was the second‐most enriched category, and within the DOWN class, it was the most enriched one (Figure 1d). Notably, 33 of the top 100 genes within the NHR‐49 UP class were included in previous studies examining gene expression changes in worms infected by the human opportunistic pathogen *Pseudomonas* *aeruginosa* (Figure S2) (Dasgupta et al., 2020; Fletcher et al., 2019; Troemel et al., 2006; Twumasi‐Boateng & Shapira, 2012) propelling us to assess NHR‐49’s role in response to this pathogen.

### 2.3 NHR‐49 contributes toward defense against P. aeruginosa infection

**FIGURE 2:**
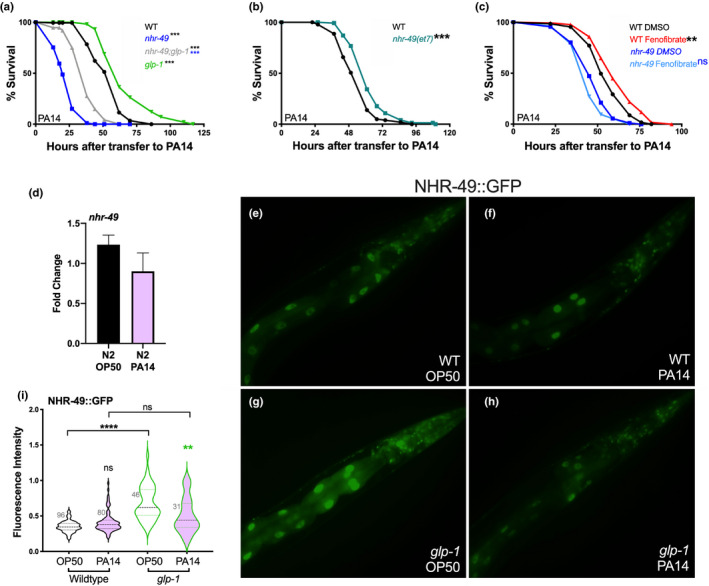
NHR‐49 contributes toward defense against *P*. *aeruginosa* pathogen attack. (a) Survival on PA14 of L4 stage wild‐type worms (WT, black, *m* = 54.03 ± 0.99, *n* = 151/198) as well as *nhr*‐*49* (blue, *m* = 23.03 ± 0.62, *n* = 184/195), *glp*‐*1* (green, *m* = 67.31 ± 1.86, *n* = 101/113) and *nhr*‐*49*;*glp*‐*1* (gray, *m* = 37.48 ± 1.02, *n* = 99/115) mutants. See Tables S3 and S4 for additional trials with these strains. (b) *nhr*‐*49 gof* mutants survives longer on PA14. Survival on PA14 of L4 stage WT worms (black; *m* = 54.32 ± 1.09, *n* = 126/145) and *nhr*‐*49(et7)* mutants (olive, *m* = 62.79 ± 1.31, *n* = 94/136). *p* < 0.0001. Data from additional trials in Table S2A. (c) Fenofibrate supplementation increases survival on PA14. Survival on PA14 of L4 stage DMSO‐control grown, WT worms (black; *m* = 56.81 ± 1.08, *n* = 103/129) and *nhr*‐*49* mutants (dark blue, *m* = 47.69 ± 0.98, *n* = 107/134) compared with Fenofibrate‐supplemented WT worms (red, *m* = 62.1 1.26, *n* = 93/135) and *nhr*‐*49* mutants (light blue, *m* = 45.33 ± 0.89, *n* = 104/134). Data from additional trials in Table S2B. (d–i) PA14 exposure reduces NHR‐49 levels. (d) *nhr*‐*49* mRNA levels measured by Q‐PCR in Day 1 adults grown on OP50 till L4 stage then transferred to PA14 plates for 8 h (pink) or continued on OP50 (black). *p* = 0.27. Data combined from three independent biological replicates, each including three technical replicates. (e–h) Representative images of NHR‐49::GFP in WT (e, f) and *glp*‐*1* mutants (g, h) grown on OP50 till L4 stage and then transferred to PA14 (f, h) or retained on OP50 (e, g) for 24 h. (i) Violin plot quantification of GFP intensity using a COPAS Biosorter. WT (black outlines), *glp*‐*1* (green outlines) exposed to PA14 (pink) or retained on OP50 (blank). Number of worms assayed shown on each panel. Data from one of three biological replicates with similar results. In (a–c), survival data shown as mean life span in hours (*m*) ± SEM (see Methods for details). In (d), error bars represent standard error of the mean (SEM). In (i), where all fluorescence measures were normalized to time‐of‐flight, the center dashed line indicates median intensity and flanking lines the first and third quartiles. Statistical significance was calculated in (a) and (b) using the log‐rank method (Mantel Cox, OASIS2), in d by using a two‐tailed *t* test, and in (i) using a one‐way nonparametric ANOVA with Dunn’s post hoc test. Statistical significance is shown on each panel next to a given strain/condition with the color of the asterisk indicating strain/condition being compared to *p* < 0.01 (**), <0.001 (***), <0.0001 (****), not significant (ns)

We tested the impact of *nhr*‐*49* inactivation on survival following infection with *P*. *aeruginosa* strain PA14 (henceforth PA14) using the Slow Killing (SK) paradigm, wherein PA14 causes *C*. *elegans* to die over the course of several days (Keith et al., 2014; Powell & Ausubel, 2008). As previously reported, *glp*‐*1* mutants survived significantly longer than wild‐type (WT) adults (Alper et al., 2010; Evans et al., 2008). However, in *nhr*‐*49*;*glp*‐*1* mutants this resistance was abrogated. *nhr*‐*49* single mutant's survival was significantly reduced on PA14 compared with WT as well (Figure 2a). We then asked if NHR‐49 hyperactivation increased immunoresistance and obtained equivocal results. Mutants carrying an NHR‐49 gain‐of‐function *(gof)* allele, *et7* (Lee et al., 2016; Svensk et al., 2013), exhibited significantly increased survival ranging from 2% to 15% in three of six trials (Figure 2b, Table S2a). Similarly, supplementation of worm food with Fenofibrate, a PPARα agonist and widely prescribed, lipid‐lowering drug (Tenenbaum & Fisman, 2012), also increased the survival of worms modestly in an *nhr*‐*49*‐dependent manner but in five of eight trials, whereas in two trials survival was reduced (Figure 2c, Table S2b). Taken together, these data suggested that NHR‐49 promoted immunoresistance against pathogenic *P*. *aeruginosa*.

### 2.4 Pathogen exposure causes reduction in NHR‐49 protein levels

*nhr*‐*49* mRNA and protein levels are both elevated in response to germline ablation (Ratnappan et al., 2014), so we asked how pathogen exposure impacted it. Using quantitative PCR (Q‐PCR), we were unable to detect any change in *nhr*‐*49* mRNA levels between worms fed the normal diet of *Escherichia coli* OP50 (OP50) versus PA14 (Figure 2d). Further, *nhr*‐*49* was not identified as a gene whose expression was altered by PA14 exposure in previous reports documenting PA14‐induced transcriptomic changes (Dasgupta et al., 2020; Fletcher et al., 2019; Troemel et al., 2006; Twumasi‐Boateng & Shapira, 2012). We examined NHR‐49 protein levels using a transgenic strain expressing GFP‐linked NHR‐49 under control of its endogenous promoter (Ratnappan et al., 2014). Visual examination did not reveal alterations in sub‐cellular localization following infection. However, a modest but widespread reduction in GFP levels was noticeable in infected animals as compared to controls. To obviate subjective bias, we performed automated quantification of fluorescence intensity using a COPAS^TM^ BIOSORT platform (Pulak, 2006). GFP levels were significantly diminished in *glp*‐*1* mutants exposed to PA14 (Figure 2e–i). In fertile animals too, PA14 infection induced a modest reduction that was visually evident but did not attain statistical significance (Figure 2e–i). Together, these data suggest that unlike GSC ablation that triggers both transcriptional and translational upregulation of NHR‐49, PA14 infection causes a modest reduction in protein levels at least in *glp*‐*1* mutants.

### 2.5 In *nhr*‐*49*;*glp*‐*1* mutants, neuronal NHR‐49 rescues immunity but longevity is rescued by expression from multiple tissues

**FIGURE 3:**
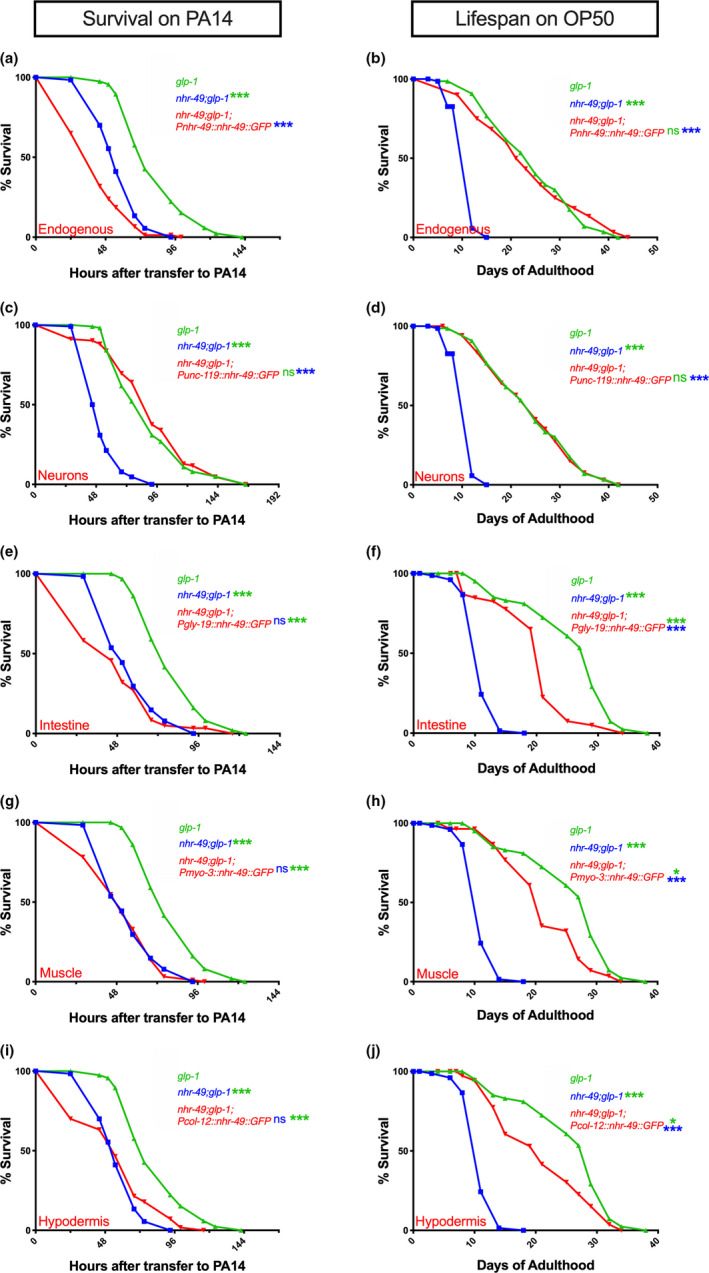
In germline‐less animals, NHR‐49 acts cell nonautonomously to promote immunity from neurons but longevity from multiple tissues. (a, c, e, g, i) NHR‐49 expression in neurons alone rescues PA14 resistance of *nhr*‐*49*;*glp*‐*1* mutants. Mean PA14 survival (in hours) of *glp*‐*1* (green), *nhr*‐*49*;*glp*‐*1* (blue), and *nhr*‐*49*;*glp*‐*1* mutants expressing NHR‐49 in different tissues (red). (a, i) Survival of *glp*‐*1* (82.88 ± 2.08, *n* = 105/120) and *nhr*‐*49*;*glp*‐*1* (57.92 ± 1.35, *n* = 108/120) mutants compared with transgenic *nhr*‐*49*;*glp*‐*1* mutants expressing NHR‐49 via Endogenous promoter (a, 43.33 ± 1.74, *n* = 95/120) or in the Hypodermis (i, 56.06 ± 2.53, *n* = 96/120). (c) Survival of *glp*‐*1* (89.65 ± 2.83, *n* = 104/108), *nhr*‐*49*;*glp*‐*1* (53.32 ± 1.26, *n* = 98/111), and *nhr*‐*49*;*glp*‐*1* mutants expressing NHR‐49 in Neurons (c, 92.09 ± 3.49, *n* = 90/102). (e, g) Survival of *glp*‐*1* (80.71 ± 1.62, *n* = 117/124), *nhr*‐*49*;*glp*‐*1* (56.25 ± 1.42, *n* = 111/121), and *nhr*‐*49*;*glp*‐*1* mutants expressing NHR‐49 in Intestine (e, 47.82 ± 2.57, *n* = 68/79) and Muscles (g, 52.44 ± 1.67, *n* = 109/115). (b, d, f, h, j) NHR‐49 expression in any somatic tissue substantially rescues longevity of *nhr*‐*49*;*glp*‐*1* mutants on OP50. Mean life span on OP50 (in days) of *glp*‐*1* (green), *nhr*‐*49*;*glp*‐*1* (blue), and *nhr*‐*49*;*glp*‐*1* mutants expressing NHR‐49 in different tissues (red). (b, d) Life span of *glp*‐*1* (24.43 ± 1.08, *n* = 51/70) and *nhr*‐*49*;*glp*‐*1* (11.28 ± 0.26, *n* = 55/72) mutants compared with transgenic *nhr*‐*49*;*glp*‐*1* mutants expressing NHR‐49 via Endogenous promoter (b, 23.72 ± 1.32, *n* = 59/60) or promoters expressed in Neurons (d, 24.41 ± 1.04, *n* = 66/71). (f, h, j) Life span of *glp*‐*1* (25.78 ± 0.98, *n* = 65/77), *nhr*‐*49*;*glp*‐*1* (11.27 ± 0.26, *n* = 72/72), and *nhr*‐*49*;*glp*‐*1* mutants expressing NHR‐49 in Intestine (f, 19.79 ± 0.95, *n* = 41/63), Muscles (h, 21.4 ± 1.02, *n* = 34/59) or Hypodermis (j, 21.06 ± 1.36, *n* = 28/42). Survival and life span data shown as mean ± standard error of the mean (SEM). “*n*” refers to number of worms analyzed over total number of worms tested in the experiment (see Methods for details). Statistical significance calculated using log‐rank (Mantel–Cox) method and indicated by asterisks on each panel next to mutant name (color of asterisk indicates strain being compared to). *p *< 0.05 (*), <0.001 (***), not significant (ns). Note: In some panels (a, i; e, g; b, d; f, h, j), assays have the same controls as they were performed in the same biological replicate. Data from additional trials and WT controls presented in Table S3a–e

NHR‐49 is widely expressed in *C*. *elegans* somatic cells (Ratnappan et al., 2014). Previously, we found that NHR‐49 expression under control of its endogenous/native promoter completely rescued the short life span of *nhr*‐*49*;*glp*‐*1* mutants to *glp*‐*1* levels when animals were fed the normal OP50 diet. We asked whether this transgene also rescued the exceptionally short survival of *nhr*‐*49*;*glp*‐*1* mutants on a PA14 pathogenic diet. Surprisingly, endogenous promoter‐driven NHR‐49 not only failed to improve the survival of *nhr*‐*49*;*glp*‐*1* mutants on PA14, it reduced it even further (Figure 3a, Table S3a). Animals carrying the same transgene showed consistent rescue of life span on OP50 (Figure 3b, Table S3a). We checked whether PA14 exposure abolished expression from the transgene explaining the lack of rescue. But, though GFP intensity was slightly reduced (as predicted above), it was widely visible in all tissues (Figure S3).

The contradictory observations with the endogenous promoter could be explained whether NHR‐49’s impact on immunoresistance is tissue specific with expression in some sites exerting pro‐immunity effects and in others reducing immunity. To test this possibility, we expressed NHR‐49 in individual tissues of *nhr*‐*49*;*glp*‐*1* mutants and examined the effect on their survival upon PA14 infection (Figure 3c,e,g,i, Table S3b–e) as well as life span on a normal OP50 diet (Figure 3d,f,h,j, Table S3b–e). We found that NHR‐49 expression selectively in the neurons of *nhr*‐*49*;*glp*‐*1* mutants (using the *unc*‐*119* promoter) (Maduro & Pilgrim, 1995) completely and reliably rescued their survival on PA14 to the same level as *glp*‐*1* mutants (Figure 3c, Table S3b). Expression in other somatic tissues had marginal and inconsistent impacts. Intestinal NHR‐49 (*gly*‐*19* promoter) (Warren et al., 2001) produced no significant increase in survival in any of three trials (Figure 3e, Table S3c), whereas hypodermal (*col*‐*12* promoter) (Pujol et al., 2008) or muscle (*myo*‐*3* promoter) (Fire & Waterston, 1989) expression showed sporadic rescues (Figure 3g,i, Table S3d,e).

We next asked how NHR‐49 expression in individual tissues (using the same promoters as above) impacted *nhr*‐*49*;*glp*‐*1* mutant's life span on OP50. Pan‐neuronal expression completely rescued life span to *glp*‐*1* levels in every trial (Figure 3d, Table S3b). Interestingly, expression in each of the other three somatic tissues also produced substantial increases in longevity (Figure 3f,h,j, Table S3c–e), although rescue to *glp*‐*1* levels was achieved by neuronal NHR‐49 alone. We tested whether these differential impacts on PA14 survival versus OP50 life span could simply be explained by differences in NHR‐49 levels or nuclear localization. The NHR‐49 tissue‐specific transgenes showed similar expression profiles and sub‐cellular localization in the different genetic backgrounds (Figure S3). We also compared the nuclear:cytoplasmic ratio of the intestinal NHR‐49::GFP transgene expressed in *nhr*‐*49*;*glp*‐*1* mutants on OP50 (longevity rescued) versus PA14 (immunosensitivity not rescued) and found no statistical difference in its sub‐cellular distribution between the two conditions either (Figure S4). Together, these experiments showed that upon GSC loss, NHR‐49 expression in individual somatic tissues rescued longevity substantially, whereas its expression in neurons alone could consistently rescue PA14 resistance.

### 2.6 In *nhr*‐*49* mutants, hypodermal NHR‐49 rescues longevity but diminishes immunity

**FIGURE 4:**
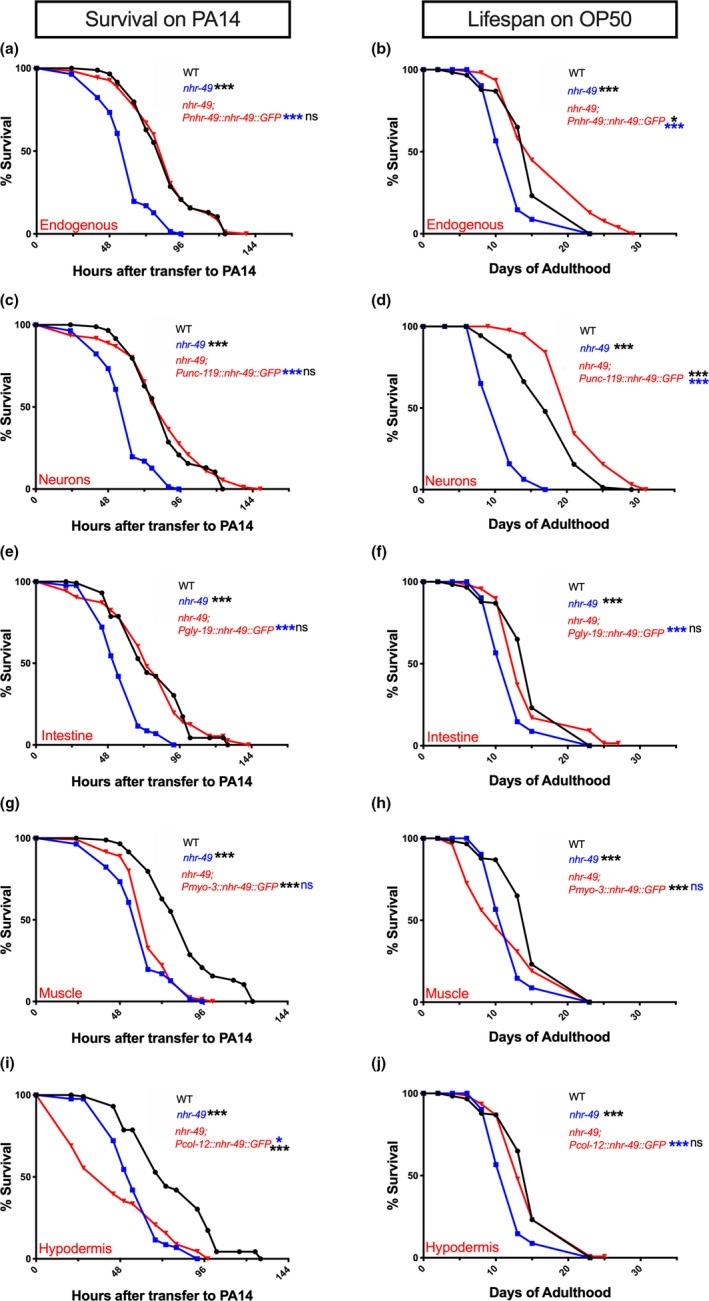
In *nhr*‐*49* mutants, neuronal NHR‐49 rescues both life span and immunity while hypodermal expression rescues longevity but lowers immunity. (a, c, e, g, i) NHR‐49 expression in neurons and intestine rescues the resistance of *nhr*‐*49* mutants on PA14. Mean PA14 survival (in hours) of WT (black, WT), *nhr*‐*49* (blue), and *nhr*‐*49* mutants expressing NHR‐49 in different tissues (red). (a, c, g) Survival of WT (84.82 ± 2.63, *n* = 55/90) and *nhr*‐*49* (60.24 ± 1.52, *n* = 102/113) strains compared to *nhr*‐*49* mutants expressing NHR‐49 via Endogenous promoter (a, 84.35 ± 2.17, *n* = 100/125) or promoters expressed in the Neurons (c, 84.44 ± 2.73, *n* = 95/110) or Muscles (g, 65.73 ± 1.27, *n* = 105/119). (e, i) Survival of WT (78.24 ± 2.57, *n* = 63/125), *nhr*‐*49* (58 ± 1.44, *n* = 99/127), and *nhr*‐*49* mutants expressing NHR‐49 in Intestine (e, 77.6 ± 2.73, *n* = 87/125) or Hypodermis (i, 46.27 ± 2.72, *n* = 79/100). (b, d, f, h, j) NHR‐49 expression in the neurons, intestine or hypodermis substantially improves *nhr*‐*49* mutant’s longevity on OP50. Mean life spans on OP50 (in days) of WT (black), *nhr*‐*49* (blue), and *nhr*‐*49* strains expressing NHR‐49 in different tissues (red). (b, f, h, j) Life span of WT (15.42 ± 0.49, *n* = 89/122) and *nhr*‐*49* (12.5 ± 0.36, *n* = 110/119) strains compared with *nhr*‐*49* mutants expressing NHR‐49 via Endogenous promoter (b, 18 ± 0.97, *n* = 97/112) or promoters expressed Intestine (f, 15.25 ± 0.4, *n* = 97/118), Muscles (h, 12.01 ± 0.71, *n* = 75/80) or Hypodermis (j, 14.86 ± 0.45, *n* = 131/141). (d) Life span of WT (17.99 ± 0.58, *n* = 71/82) and *nhr*‐*49* (11.11 ± 0.33, *n* = 63/65) strains compared with *nhr*‐*49* mutants expressing NHR‐49 in Neurons (d, 22.23 ± 0.71, *n* = 33/76). Survival and life span data shown as mean ± standard error of the mean (SEM). “*n*” refers to number of worms analyzed over total number of worms tested in the experiment (see Methods for details). Statistical significance calculated using log‐rank (Mantel–Cox) method and indicated by asterisks on each panel next to mutant name (color of asterisk indicates strain being compared to). *p *< 0.05 (*), <0.001 (***), not significant (ns). Note: assays in some panels (a, c, g; e, i; b, f, h, j) have the same controls as they were performed in the same biological replicate. Data from additional trials and wild‐type controls presented in Table S4a–e

Since *nhr*‐*49* single mutants also exhibit shortened survival compared with WT, we investigated which tissues NHR‐49 acted in to promote their survival on PA14 and OP50. However, unlike in the *nhr*‐*49*;*glp*‐*1* background, the endogenous promoter‐driven NHR‐49 transgene rescued the survival of *nhr*‐*49* single mutants reliably on PA14 (98% rescue in 3/4 trials, Figure 4a, Table S4a) as well as on OP50. In fact, life span on OP50 was augmented even further than WT as observed in our previous work (Figure 4b, Table S4a) (Ratnappan et al., 2014). As in *nhr*‐*49*;*glp*‐*1* mutants, pan‐neuronal expression completely rescued both immunoresistance and life span of *nhr*‐*49* mutants (Figure 4c,d, Table S4b). Intestinal expression also significantly rescued both longevity and immunity (Figure 4e,f, Table S4c), whereas muscle expression rescued neither (Figure 4g,h, Table S4d). Strikingly though, hypodermal NHR‐49 completely rescued longevity on OP50, but survival on PA14 was significantly worsened (≥19% reduction in 4/4 trials, Figure 4i,j, Table S4e). These results, along with the observations above, demonstrate that NHR‐49 acts cell nonautonomously to modulate both longevity and immunity.

### 2.7 In WT animals, elevating NHR‐49 levels in neurons, or intestine, enhances immunoresistance

**FIGURE 5:**
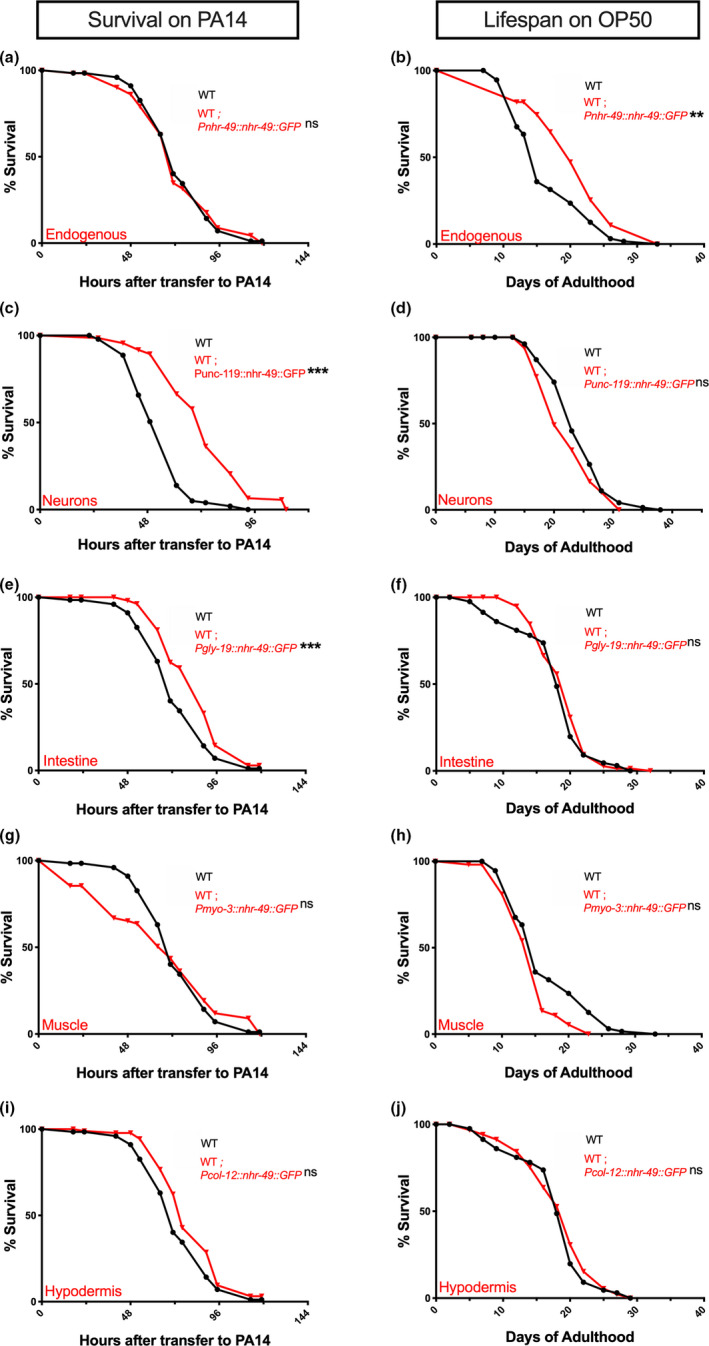
In wild‐type animals, elevating NHR‐49 levels in neurons or intestine enhances immunity. (a, c, e, g, i) NHR‐49 overexpression in neurons or intestine increases immunity. Mean survival on PA14 (in hours) of WT worms (black) and strains overexpressing NHR‐49 in different tissues (red). (a, e, i, g) Survival of WT (76.98 ± 2.25, *n* = 102/129) and strains overexpressing NHR‐49 via Endogenous promoter (a, 76.89 ± 3.06, *n* = 75/121) or promoters expressed in Intestine (e, 91.97 ± 2.65, *n* = 96/124), Muscles (g, 72.11 ± 4.28, *n* = 66/100), or Hypodermis (i, 82.68 ± 2.65, *n* = 63/97). (c) Survival of WT (54.32 ± 1.09, *n* = 125/145) and strain overexpressing NHR‐49 in Neurons (c, 73.72 ± 1.61, *n* = 114/136). (b, d, f, h, j) NHR‐49 upregulation in individual somatic tissues does not enhance immunity. Mean life span on OP50 (in days) of WT worms (black) and strains overexpressing NHR‐49 in different tissues (red). (b, h) Life span of WT (16.67 ± 0.65, *n* = 67/73) and strains overexpressing NHR‐49 via Endogenous promoter (b, 20.83 ± 1.01, *n* = 35/44) or promoter expressed in Muscles (h, 14.6 ± 0.55, *n* = 39/52). (d) Life span of WT (24.2 ± 0.55, *n* = 74/91) and strain overexpressing NHR‐49 in Neurons (d, 22.35 ± 0.6, *n* = 57/94). (f, j) Life span of WT (17.81 ± 0.56, *n* = 73/121) and strains overexpressing NHR‐49 in Intestine (f, 19.06 ± 0.4, *n* = 86/116) or Hypodermis (j, 18.3 ± 0.53, *n* = 95/120). Survival and life span data shown as mean ± standard error of the mean (SEM). “*n*” refers to number of worms analyzed over total number of worms tested in the experiment (see Methods for details). Statistical significance calculated using log‐rank (Mantel–Cox) method and indicated by asterisks on each panel next to mutant name (color of asterisk indicates strain being compared to). *p* < 0.01 (**), <0.001 (***), not significant (ns). Note: assays in some panels (a, i, e, g; b, h; f, j) have the same controls as they were performed in the same biological replicate. Data from additional trials are presented in Table S5a–e

NHR‐49 protein levels are important in determining the animals’ life span because elevating its levels in normal, fertile adults, either using the endogenous promoter or in neurons alone, has been reported to induce a modest life span extension (Burkewitz et al., 2015; Ratnappan et al., 2014). We asked whether endogenous promoter‐driven overexpression increased PA14 resistance as well but observed ambivalent effects as survival was increased in only 1/3 trials (Figure 5a,b Table S5a). We next asked whether elevating NHR‐49 levels in individual tissues could enhance immunoresistance. WT animals’ immunity was enhanced by NHR‐49 overexpression in the neurons or the intestine (~15%–30%) (Figure 5c,e, Table S5b,c, respectively), whereas in the muscles or hypodermis it did not produce consistent impacts (Figure 5g,i, Table S5d,e). Intriguingly, the benefits obtained by intestinal or neuronal upregulation were restricted to survival during PA14 infection. We did not observe a consistent life span extension on OP50 when NHR‐49 was overexpressed in any single somatic tissue (Figure 5d,f,h,j, Table S5b–e).

**FIGURE 6:**
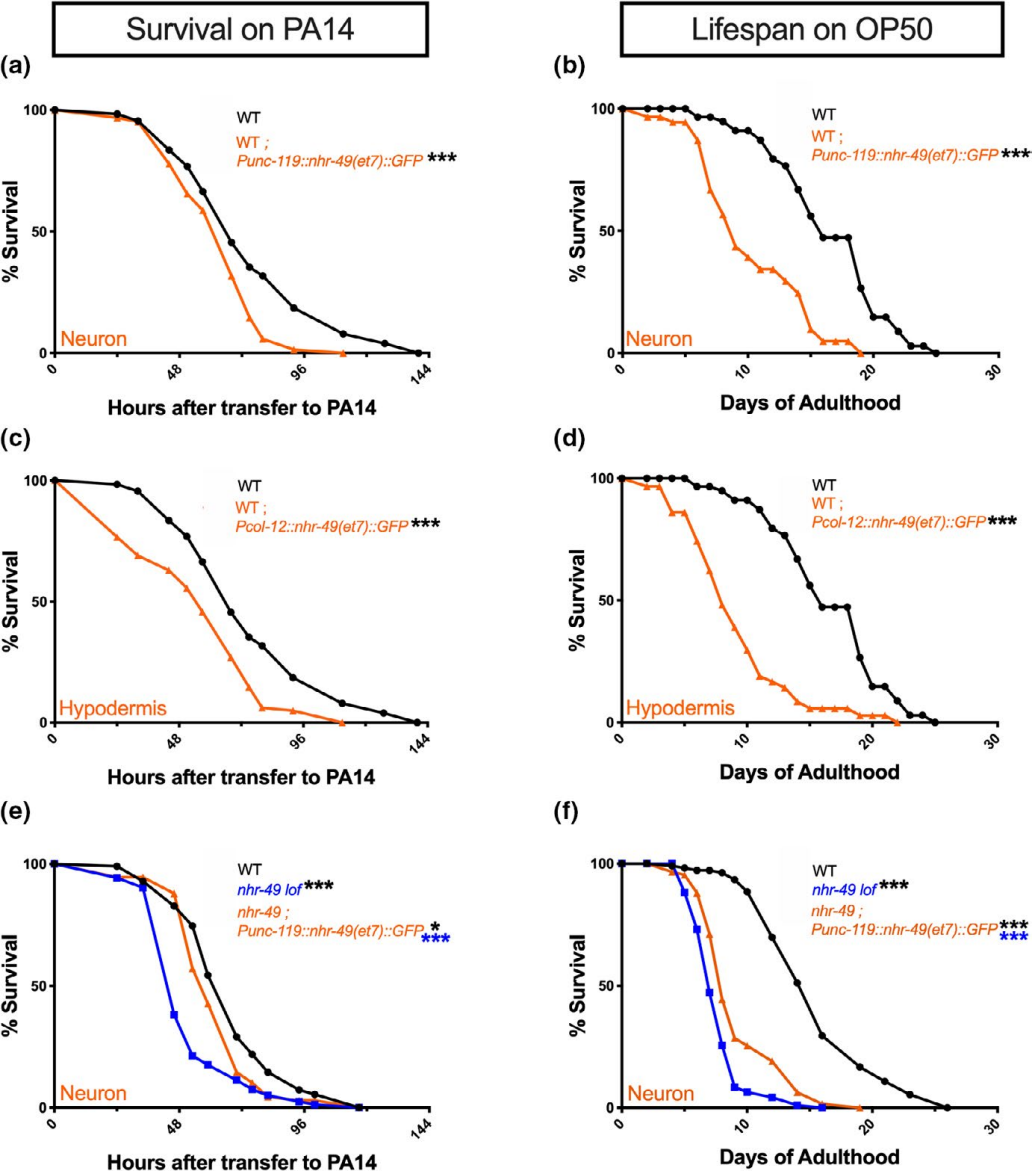
Expression of NHR‐49*^et7^* in neurons or hypodermis reduces immunoresistance and longevity. (a–d) NHR‐49*^et7^* in neurons (a, b) or hypodermis (c, d) of wild‐type (WT) worms reduces survival upon PA14 exposure (a, c) and life span on OP50 (b, d). Mean survival on PA14 (in hours) of WT (black) and NHR‐49*^et7^* transgenic strains (orange). (a, c) WT (75.15 ± 2.74, *n* = 94/120), NHR‐49*^et7^* expressed in Neurons (a, 62.92 ± 1.59, *n* = 98/125) or Hypodermis (c, 55.46 ± 2.11, *n* = 118/145). (b, d) Mean life span on OP50 (in days) of WT (black) and NHR‐49*^et7^* transgenic strains (orange). WT (16.49 ± 0.44, *n* = 103/136), NHR‐49*^et7^* expressed in Neurons (b, 10.23 ± 0.74, *n* = 28/60) or Hypodermis (d, 9.09 ± 0.53, *n* = 60/90). (e, f) Neuronal expression of NHR‐49*^et7^* partially rescues immunosensitivity of *nhr*‐*49* mutants. (e) Survival on PA14 (in hours) of WT (black, 67.46 ± 2.15, *n* = 78/106), *nhr*‐*49* (blue, 52.23 ± 1.69, *n* = 94/105), and *nhr*‐*49* mutants expressing NHR‐49*^et7^* in Neurons (orange, 61.77 ± 1.71, *n* = 89/111). (f) Life span on OP50 (in days) of WT (black, 15.61 ± 0.46, *n* = 99/120), *nhr*‐*49* (blue, 7.66 ± 0.2, *n* = 101/119), and *nhr*‐*49* mutants expressing NHR‐49*^et7^* in Neurons (orange, 9.31 ± 0.38, *n* = 69/89). Survival and life span data shown as mean ± standard error of the mean (SEM). “*n*” refers to number of worms analyzed over total number of worms tested in the experiment (see Methods for details). Statistical significance was calculated using the log‐rank (Mantel–Cox) method and is indicated by asterisks on each panel next to mutant name (color of asterisk indicates strain being compared to). *p *< 0.05 (*), <0.001 (***). Note: assays in some panels (a, c; b, d) have the same controls as they were performed in the same biological replicate. Data from additional trials presented in Table S6

NHR‐49 undergoes lipid ligand‐dependent activation (Folick et al., 2015), so one explanation for its tissue‐specific effects could be differential activation, that is, overexpression is not sufficient, because it is only activated in tissues where it promotes immunity or longevity but not in others. We tested this possibility by expressing the *et7 gof* allele (NHR‐49*^et7^*) (Lee et al., 2016; Svensk et al., 2013) in the hypodermis (where its expression had no beneficial impact) or in neurons (where its expression had consistently beneficial effects). Surprisingly, we found that either neuronal or hypodermal expression of NHR‐49*^et7^* drastically shortened survival on both OP50 and PA14 (Figure 6a–d, Table S6a). Neuronal NHR‐49*^et7^* expression produced a small but significant rescue of *nhr*‐*49* mutants’ immunity and longevity phenotypes (Figure 6e,f; Table S6a). We could not create viable strains that expressed hypodermal NHR‐49*^et7^* in any *nhr*‐*49* mutant background suggesting that unfettered NHR‐49 activation may in fact have severe adverse consequences. We also tested whether chemical activation of NHR‐49 by Fenofibrate supplementation in strains expressing NHR‐49 in muscles or hypodermis of *nhr*‐*49* mutants (where no rescue was observed) improved survival on PA14 but observed no rescue in either strain (Table S6b).

### 2.8 In *glp*‐*1* mutants, elevating NHR‐49 levels in somatic tissues does not further enhance immunoresistance

Lastly, we assessed the consequences of raising NHR‐49 levels in animals that have elevated protein to begin with, that is, in *glp*‐*1* mutants wherein NHR‐49 is both transcriptionally and translationally upregulated (Ratnappan et al., 2014). Further overexpression in this genetic background, either using the widespread endogenous promoter or tissue‐specific drivers, did not enhance either longevity or immunity further. In fact, it appeared to diminish survival, especially upon PA14 and in some cases on OP50 as well (Figure S5a–j, Table S7a–e). Altogether, our site‐of‐action experiments substantiated the importance of not only the location and levels of NHR‐49 but also its tissue‐specific activation in determining the impact on life span versus immune status of the animal.

### 2.9 *fmo*‐*2* and *acs*‐*2* are differentially impacted by germline loss versus pathogen attack

**FIGURE 7:**
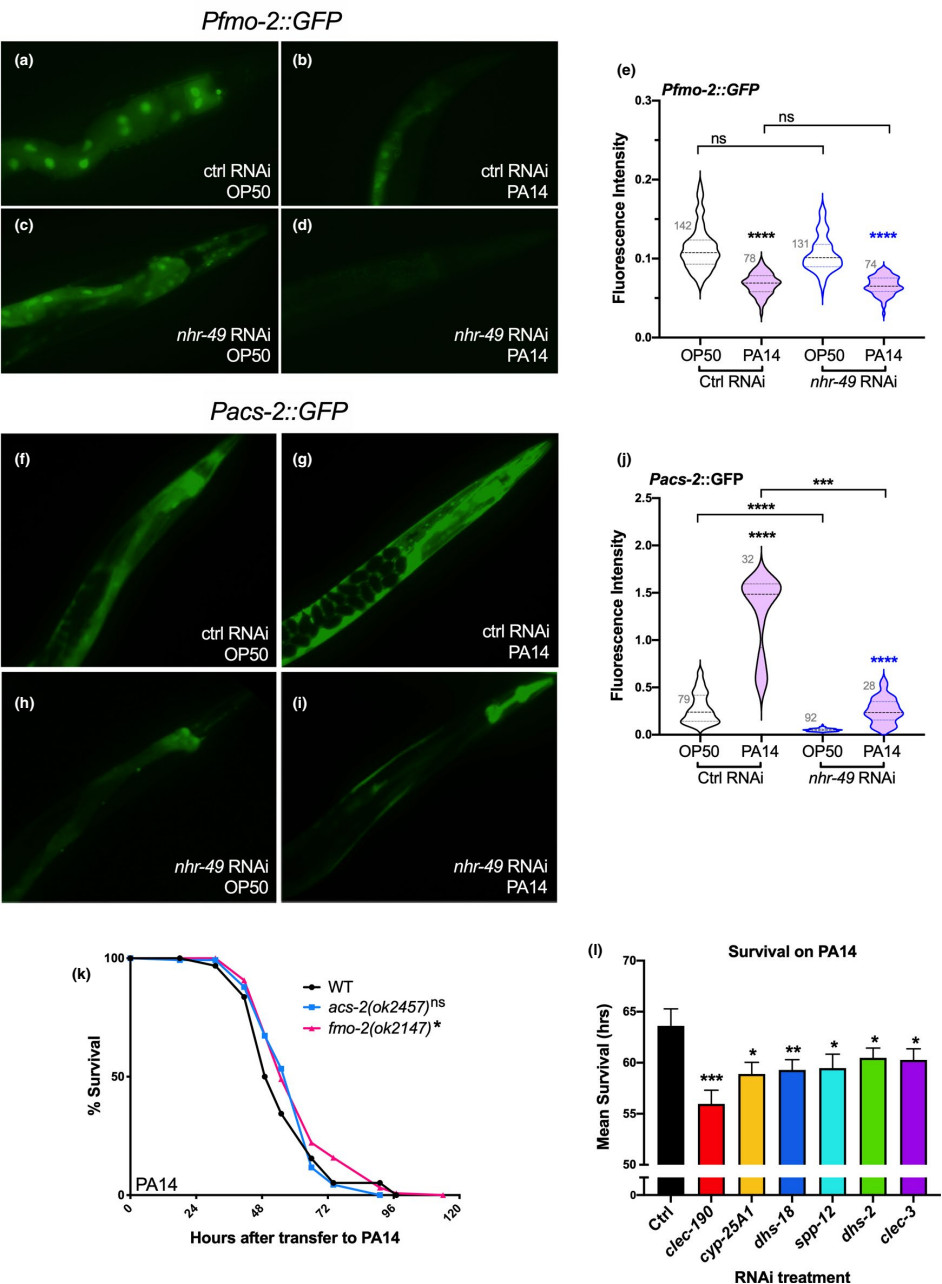
Known NHR‐49 targets, *fmo*‐*2* and *acs*‐*2*, do not contribute toward PA14 resistance. (a–e) *fmo*‐*2* expression decreases upon PA14 exposure in an *nhr*‐*49*‐independent manner. (a–d) Representative GFP images of Day 2 P*fmo*‐*2p*::GFP adults grown till Day 1 on vector control (ctrl, a, b) or *nhr*‐*49* RNAi (c, d) bacteria before transfer to PA14 (b, d) or OP50 (a, c) for 24 h. (e) Violin plot showing quantification of GFP intensity using a COPAS Biosorter. Vector control (Ctrl, black outline) or *nhr*‐*49* RNAi (blue outline). PA14 exposure (pink) or OP50 (blank). Number of worms assayed per condition shown on panel. Data from one of three trials that gave similar results. (f–j) *acs*‐*2* expression increases modestly upon PA14 exposure in an *nhr*‐*49*‐dependent manner. (f–i) Representative GFP images of Day 2 P*acs*‐*2p*::GFP adults grown till Day 1 on vector control (ctrl, a, b) or *nhr*‐*49* RNAi (c, d) bacteria before transfer to PA14 (b, d) or OP50 (a, c) for 24 h. (j) Violin plot showing quantification of GFP intensity using a COPAS Biosorter. Vector control (Ctrl, black outline) or *nhr*‐*49* RNAi (blue outline). PA14 exposure (pink) or OP50 (blank). Number of worms assayed per condition shown on panel. (k) Survival of L4 stage wild‐type worms (WT, black) and *acs*‐*2* (blue), and *fmo*‐*2* (pink) mutants exposed to PA14. WT (*m* = 56.66 ± 1.4, *n* = 97/127), *acs*‐*2* (*m* = 59.52 ± 1.19, *n* = 94/137), and *fmo*‐*2* (*m* = 62.53 ± 1.36, *n* = 136/153). (l) NHR‐49 target genes encoding anti‐microbial proteins contribute toward PA14 resistance. Bar graph representation of mean survival (in hours) of wild‐type animals exposed till L4 stage to control vector (Ctrl) or RNAi clones targeting NHR‐49 target genes (see Methods for details). In (e) and (j), center dashed line indicates median intensity and lines flanking it represent the first and third quartiles; data from one of three trials that gave similar results shown. In (k) and (l), survival data shown as mean life span in hours (*m*) ± SEM (see Methods for details). Statistical significance was calculated in (e) and (j) using a one‐way nonparametric ANOVA with Dunn’s post hoc test (Graphpad Prism). Significance was calculated in (k) and (l) using the log‐rank method (Mantel Cox, OASIS2). Statistical significance shown on each panel with the color of the asterisk indicating the strain being compared to. *p* <0.05 (*), < 0.01 (**), <0.001 (***), <0.0001 (****), not significant (ns)

We asked whether the differential impacts of NHR‐49 on longevity versus immunity extended to expression of its downstream targets too. Our NHR‐49 UP group included the well‐established *nhr*‐*49*‐target gene, *acs*‐*2*, that encodes an acyl CoA synthetase involved in mitochondrial β‐oxidation (Van Gilst, Hadjivassiliou, Jolly, et al., 2005; Van Gilst, Hadjivassiliou, & Yamamoto, 2005) and is upregulated in *glp*‐*1* mutants (Amrit et al., 2016), as well as *fmo*‐*2*, that encodes a flavin monooxygenase (Table S1a) (Goh et al., 2018; Leiser et al., 2015). Recently, both genes have been reported to be dramatically upregulated upon infection by the gram‐positive pathogens, *Enterococcus faecalis*, and *Staphylococcus aureus* (Dasgupta et al., 2020; Wani et al., 2021). We tested whether PA14 exposure altered their expression as well. Instead, *Pfmo*‐*2p*::*GFP* was significantly downregulated upon PA14 exposure and independent of NHR‐49 activity (Figure 7a–e). *Pacs*‐*2p*::*GFP* showed a small, NHR‐49‐dependent increase in expression on PA14 (Figure 7f–j). Both genes have been reported to be essential for survival during *E*. *faecalis* infection, and *fmo*‐*2* mutants are also hyper‐susceptible to *S*. *aureus* infection (Dasgupta et al., 2020; Wani et al., 2021). But, mutants of neither gene showed reduced survival upon PA14 exposure (Figure 7k).

### 2.10 NHR‐49 targets encoding antimicrobial proteins play roles in *Pseudomonas* resistance

In addition to *acs*‐*2*, we had previously identified numerous other genes involved in fatty acid β‐oxidation that are upregulated in, and contribute to the longevity of, *glp*‐*1* mutants, dependent upon NHR‐49, DAF‐16 and TCER‐1 (Amrit et al., 2016; Ratnappan et al., 2014). Accordingly, our NHR‐49 UP group included 24 other genes with roles in β‐oxidation and lipid hydrolysis (Table S8). Of these, only three were identified as being upregulated upon PA14 exposure, whereas seven were in fact downregulated (Table S8) (Dasgupta et al., 2020; Fletcher et al., 2019; Troemel et al., 2006; Twumasi‐Boateng & Shapira, 2012). Upon testing the impact of RNAi inactivation of two of these genes, *acox*‐*1*.*1* (upregulated in both conditions), or *hacd*‐*1* (upregulated in *glp*‐*1*, downregulated on PA14), we observed no consistent change in survival during PA14 exposure (Table S9). The NHR‐49 UP class also included multiple members of families encoding antimicrobial proteins (saposins, C‐type lectins) and xenobiotic‐metabolizing enzymes such as cytochrome P450s and short‐chain dehydrogenases (Table S1a) (Menzel et al., 2007). We found that RNAi inactivation of six of eight genes encoding these proteins (*clec*‐*190*, *clec*‐*3*, *dhs*‐*18*, *dhs*‐*2*, *spp*‐*12*, *spp*‐*9*, *cyp*‐*14A3*, and *cyp*‐*25A1*) (Figure 7l, Figure S6a,b) diminished PA14 resistance of normal worms suggesting that the anti‐microbial proteins and xenobiotic‐neutralizing enzymes directed by NHR‐49 may have functional roles in defense against PA14 infection.

## 3 DISCUSSION

In this study, we demonstrate a role for NHR‐49 in innate immunity and provide multiple lines of evidence that, although NHR‐49 confers both pathogen resistance and long life, it modulates these processes distinctly. We show that these distinctions (a) arise from differential regulation of NHR‐49 by a life span‐altering intervention such as germline loss versus the acute stress of pathogen attack, (b) extend to discrete sites of action and transcriptional outputs, and (c) culminate in differential functional roles of downstream target genes.

### 3.1 In biology, context is critical

In worms and other species, numerous studies have established the cell nonautonomous regulation of longevity and stress response pathways and identified tissues where key factors act to modulate these processes (Dillin et al., 2014; Medkour et al., 2016; Morimoto, 2020). Neuron‐expressed factors that mediate pathogen recognition and avoidance as well as systemic induction of antimicrobial gene expression have been elucidated as well (Hoffman & Aballay, 2019; Wani et al., 2020). But, in addition to site‐of‐expression, physiological context is crucial in determining whether a protein has beneficial, benign, or detrimental impacts. A regulatory factor may act in different tissues to modulate different biological processes (Kodani & Nakae, 2020). Or, within the same tissue, the activity of a protein may have diametrically opposite effects on different aspects of health (Amrit et al., 2019). The cell nonautonomous regulation of longevity versus stress resistance has been compared for few factors such as DAF‐16 (Libina et al., 2003), XBP‐1 (Taylor et al., 2014; Taylor & Dillin, 2013) and TCER‐1 (Amrit et al., 2019). By documenting the impact of NHR‐49 expression in each of five different tissues, in each of four genetic backgrounds, on longevity as well as immunity, our study provides substantive evidence on the importance of physiological context in determining gene function.

We found that neuronal NHR‐49 alone could consistently rescue the immunity deficits of *nhr*‐*49*;*glp*‐*1* mutants, whereas their life span was substantially restored by expression in any somatic tissue. In *nhr*‐*49* single mutants, immunity was restored by presence in neurons or intestine, but life span could be rescued from other tissues as well. This suggests that pathogen response may be more sensitive to NHR‐49’s location as compared to longevity. Interestingly, NHR‐49 expression in muscles provided little or no immunity benefit in any genetic background we tested. In fact, expression in muscles mostly diminished immunity, in contrast to the broad immunity advantages conferred by neuronal NHR‐49. Of note, similar observations have been made with the endoplasmic reticulum unfolded protein response (UPR^er^) regulator, XBP1, whose expression in neurons or intestine increases life span and proteostasis but expression in muscles diminishes both (Imanikia et al., 2019). Another intriguing observation in our study is the impact of endogenous promoter‐driven NHR‐49 expression on the PA14 resistance of *nhr*‐*49*;*glp*‐*1* mutants. Not only was this transgene unable to rescue the mutants’ PA14 sensitivity, it further diminished their survival. While it is possible that this is simply a consequence of transgene toxicity, it is unlikely because it is a functional transgene that (a) completely rescued the PA14 resistance of *nhr*‐*49* mutants (b) completely rescued the life span on OP50 of both *nhr*‐*49*;*glp*‐*1* and *nhr*‐*49* mutants (c) did not cause life span shortening in WT animals fed OP50 or PA14, and in fact extended life span on OP50. These data further emphasize that pathogen resistance is exquisitely sensitive to NHR‐49’s site‐ and level‐ of expression, and modest changes in either can have major consequences for the animal's immunity.

### 3.2 Stress and pathogen‐specific activities of NHR‐49

NHR‐49 mediates defense against pathogens such as *E*. *faecalis* and *S*. *auerus* (Dasgupta et al., 2020; Wani et al., 2021). Together with these reports, our data suggest that it orchestrates pathogen‐specific transcriptional and functional outputs. For instance, *acs*‐*2* and *fmo*‐*2* are upregulated >10,000 fold and ~150 fold, respectively, upon *S*. *aureus* infection, and on *E*. *faecalis*, *acs*‐*2* is elevated >1,000 fold (Dasgupta et al., 2020; Wani et al., 2021). But, we found *fmo*‐*2* to be downregulated by PA14 infection, whereas *acs*‐*2* showed a small increase. While these genes are critical for survival upon *E*. *faecalis* (*acs*‐*2* and *fmo*‐*2*) (Dasgupta et al., 2020) or *S*. *aureus* (*fmo*‐*2*) (Wani et al., 2021) infection, neither one contributed toward PA14 resistance or *glp*‐*1* longevity (Figure S6c). *fmo*‐*2* shows similarly specific roles in other stress paradigms as mutants are sensitive to starvation but resistant against oxidative stress (Goh et al., 2018). Interestingly, NHR‐49 also appears to differentially regulate β‐oxidation and lipid hydrolysis genes between GSC loss (Ratnappan et al., 2014), other stressors (Goh et al., 2018; Svensk et al., 2013; Van Gilst, Hadjivassiliou, Jolly, et al., 2005) and pathogenic infections (Dasgupta et al., 2020; Wani et al., 2021). Few lipolytic NHR‐49 targets elevated in *glp*‐*1* mutants appear to be induced by PA14 infection (Table S8). The inactivation of two such genes did not reduce PA14 resistance in our study. However, considering the limitations of feeding RNAi, the broader relevance of NHR‐49‐driven lipid metabolic changes in PA14 response remains to be investigated.

Why does NHR‐49 expression in different tissues impart distinctive effects on immunity and longevity? As with any array‐based transgene study, the possibility that these effects were produced by nonphysiological NHR‐49 overexpression cannot be overruled. However, we believe this is unlikely because our conclusions are based on the differential rescue of longevity versus immunity phenotypes conferred by the same transgene in a given mutant genetic background. Further, introducing these transgenes into tissues of WT animals (with physiological levels of NHR‐49) or *glp*‐*1* mutants (that had elevated NHR‐49) did not produce the same effects suggesting that simply elevating NHR‐49 levels is not sufficient. In fact, expression in the *glp*‐*1* background appeared to be widely detrimental. We did not see differences in NHR‐49 sub‐cellular localization too, and our experiments with NHR‐49*^et7^* and Fenofibrate suggest that tissue‐specific activation may not be the major determinant either. It can possibly be attributed to the presence of tissue‐specific cofactors that help orchestrate local expression profiles. DAF‐16 in neurons relies on FKH‐9 to drive expression of memory and axon regeneration genes (Kaletsky et al., 2016), whereas its intestinal transcriptome is shared with PQM‐1 (Tepper et al., 2013). NHR‐49 partners with NHR‐80 and NHR‐13 to regulate fatty acid desaturation (Folick et al., 2015; Goudeau et al., 2011; Pathare et al., 2012), and with NHR‐71 to modulate germline‐less longevity, respectively (Ratnappan et al., 2016). Of these, we found only *nhr*‐*80* RNAi to induce a small reduction in survival on PA14 (Table S9). The roles of NHR‐71 and NHR‐13 cannot be obviated though due to the ambiguities associated with feeding RNAi. Other NHR‐49 partners, including 11 NHRs that promote *glp*‐*1* longevity (Ratnappan et al., 2016), may also serve as tissue‐specific co‐regulators. Like NHR‐49, PPARα has roles in starvation‐induced fatty acid oxidation, oxidative stress, heat resistance, inflammation and immunotolerance against commensal gut microbiota (Bougarne et al., 2018; Christofides et al., 2020; Manoharan et al., 2016). It will be interesting to ask whether the immunity‐promoting function of NHR‐49 also extends to mammalian PPARα.

## 4 MATERIALS AND METHODS

### 4.1 *C*. *elegans* strains and life span assays

All strains were maintained by standard techniques at 20℃ or 15℃ on nematode growth medium (NGM) plates seeded with an *E*. *coli* strain OP50. For experiments involving RNAi, NGM plates were supplemented with 1 ml 100 mg/ml Ampicillin and 1 ml 1 M IPTG (Isopropyl β‐D‐1‐thiogalactopyranoside) per liter of NGM. The main strains used in this study include N2 (wild type), CF1903 *[glp*‐*1(e2144)* *III]*, AGP12a *[nhr*‐*49(nr2041)I]*, AGP22 [*nhr*‐*49(nr2041)I*;*glp*‐*1(e2141)III]*, AGP110 *[nhr*‐*49(et7)I]*. All strains listed in Table S10. Life span experiments were performed as previously described (Amrit et al., 2014). For life span in the *glp*‐*1* background, eggs were kept at 20℃ for 2–6 h, grown to the L4 stage at 25℃, then shifted back to 20℃ for remaining life span. In life span assays, the L4 stage was counted as Day 0 of adulthood. Fertile strains were transferred to fresh plates every other day to separate parents from progeny. Animals that exploded, bagged, crawled off the plate, or became contaminated were marked as censored upon observation. The program Online Application of Survival Analysis 2 (OASIS 2) (Han et al., 2016) was used for statistical analysis of both life span and pathogen stress assays. *p*‐Values were calculated using the log‐rank (Mantel–Cox) test (Han et al., 2016). Results were graphed using GraphPad Prism (version 8).

### 4.2 Pathogen survival assays

For survival assays on PA14, Luria Bertani (LB) agar plates were streaked with PA14 bacteria from −80℃ glycerol stocks, incubated at 37℃ overnight and stored at 4℃ for ≤1 week. Single PA14 colonies from streaked plates were then inoculated into 3 ml King’s Broth (Sigma) overnight (16–18 h) in a 37℃ shaking incubator. 20 μl of this culture was seeded onto slow‐killing (NGM with 0.35% peptone) plates and incubated at 37℃ for 24 h (Keith et al., 2014; Tan & Ausubel, 2000). Seeded PA14 plates were kept at room temperature for 24 h before use. PA14 survival assays were performed as previously described (Keith et al., 2014). Age‐matched *C*. *elegans* strains were grown under the same conditions and selected at the L4 stage as for the life span experiments. 25–30 L4 worms per plate were transferred to each of five PA14 plates per strain and maintained at 25℃ till the end of their lives. Strains were monitored at 6–12 h intervals to count living, dead, and censored animals as described above. Living animals were transferred to fresh PA14 plates each day for 3–4 days. Statistical analysis of survival data was performed on OASIS 2, and representative trials were graphed with GraphPad Prism (version 8). For RNAi experiments, the same protocol was followed except for growing worms from egg‐to‐L4 stage on plates seeded with bacteria (HT115) containing RNAi constructs or empty vector control (pAD12).

### 4.3 RNA sequencing and data analysis

RNA was isolated from three biological replicates of Day 2 adults of CF1903 (*glp*‐*1)* and AGP22 (*nhr*‐*49*;*glp*‐*1)* strains, grown as described above. Following 7 freeze thaw cycles, approximately 3,000 worms were harvested for RNA using the TRIzol method. RNA was checked for quality and quantity using the Agilent Tapestation and Qubit Fluorometry. Sequencing libraries were prepared using the TruSeq stranded mRNA (PolyA+) kit, and the samples were then subjected to 75 base pair paired‐end sequencing on an Illumina NextSeq 500 sequencer at the Univ. of Pittsburgh Genomics Research Core. Sequencing data were analyzed using the CLC Genomics Workbench (Version 20.0.3) employing the RNA‐Seq pipeline. Differentially regulated genes were filtered for significant changes based on the criteria of >2 fold change in expression, *p* Value of <0.05 and a false discovery rate (FDR) of <0.05. The raw RNAseq data has been uploaded to GEO and is available with accession series GSE158729.

### 4.4 Gene Ontology analyses

Genes that were differentially regulated in a statistically significant manner were classified into two groups as either upregulated (UP) or downregulated (DOWN) NHR‐49 targets. These groups were analyzed for enrichment of gene classes based on Gene Ontology (GO) terms using *C*. *elegans* centered publicly available online resources, Wormbase Gene Set Enrichment Analysis tool (https://wormbase.org/tools/enrichment/tea/tea.cgi) and WormCat (http://wormcat.com/) (Holdorf et al., 2020). Representation Factor was calculated at http://nemates.org/MA/progs/overlap_stats.html.

### 4.5 Quantitative PCRs

RNA was isolated as mentioned above, quantified using a Nanodrop and DNAse treated (DNAse kit, Sigma AMPD1). The RNA was then reverse transcribed into cDNA using the High‐Capacity cDNA Reverse Transcription kit (Applied Biosystems, 4368814) following the manufacturer's recommendations. RNA samples were collected from three independently isolated “biological replicates,” and three “technical replicates” of each strain/condition were tested for a given biological replicate. Quantitative PCRs were conducted using the PowerUp SYBR Green Master Mix kit (Applied Biosystems A25741) on the CFX Connect Machine (BioRad). Gene expression data were analyzed using the ΔΔ*C*_t_ method and normalized to the housekeeping gene, *rpl*‐*32*. Melt curves for all reactions were run confirming the integrity of the reaction. Primer sequences are included in Table S11.

### 4.6 Transgenic strain generation

Tissue‐specific NHR‐49 expressing strains were generated by plasmid microinjection. A control P*nhr*‐*49*::*nhr*‐*49*::*gfp* (pAG4) construct, which drives NHR‐49 expression via its endogenous promoter, was created as previously described (Ratnappan et al., 2014). To drive NHR‐49 expression in other tissues, 4.4 kb coding region of *nhr*‐*49* was first amplified with modified primers to introduce SbfI and SalI restriction sites at the 5′ end and SmaI at the 3′ end of the coding region. This product was cloned into the GFP expression vector pPD95.77 (Addgene plasmid 1495) upstream of, and in frame with, GFP. Individual tissue‐specific promoters were then amplified and ligated independently into this plasmid using primers modified with the forward primer including SbfI and the reverse including SalI to create plasmids for expressing NHR‐49 in the muscle (P*myo*‐*3*::NHR‐49::GFP), intestine (P*gly*‐*19*::NHR‐49::GFP), hypodermis (P*col*‐*12*::NHR‐49::GFP), and neurons (P*unc*‐*119*::NHR‐49::GFP). Each of the five constructs was then injected at a concentration of 100 with 15 ng/mL of P*myo*‐*2*::*mCherry* co‐injection marker into *nhr*‐*49*, *nhr*‐*49*;*glp*‐*1*, *glp*‐*1*, and WT animals to create transgenic strains carrying the individual extragenic arrays. The *et7* mutation (C > T) was introduced into *Punc*‐*119*::*nhr*‐*49*::*GFP* and *Pcol*‐*12*::*nhr*‐*49*::*GFP* plasmids using the Q5 Site‐Directed Mutagenesis Kit from (New England Biolabs, E0554S). Plasmids were sequenced to confirm the presence of the *et7* mutation and then injected into WT, *nhr*‐*49* and *nhr*‐*49*;*glp*‐*1* animals as described above. No viable transgenics were obtained for *nhr*‐*49* mutants expressing *Pcol*‐*12*::*nhr*‐*49*::*GFP* and *nhr*‐*49*;*glp*‐*1* *mutants* expressing either *Punc*‐*119*::*nhr*‐*49*::*GFP* or *Pcol*‐*12*::*nhr*‐*49*::*GFP*. For each of the 23 transgenic strains generated in this study, 2–4 independent transgenic lines were generated. Transgene‐carrying strains were maintained and selected for life span and pathogen stress assays using a Leica M165C microscope with a fluorescence attachment. A complete listing of all strains used in this study is provided in Table S10, and primer sequences are in Table S11.

### 4.7 Fenofibrate supplementation assay

100 μL of 10 μM Fenofibrate (Sigma F6020) in 0.1% DMSO was placed onto both NGM and slow‐killing plates before seeding with OP50 or PA14, respectively, as described above (Brandstädt et al., 2013; Leiteritz et al., 2020). Upon the drying and growth of the bacterial lawn, eggs were grown to L4 on either the Fenofibrate or 0.1% DMSO control plates and then were transferred to PA14 plates (similarly supplemented with Fibrate or DMSO) at L4 larval stage and survival monitored. Worms were transferred to fresh plates as described above.

### 4.8 GFP fluorescence imaging and quantitation

GFP expression in transgenic strains was quantified using the COPAS Biosorter (Union Biometrica) as described in Pujol et al. (2008). For setup, progeny of transgenic mothers were grown to the Day 1 of adulthood (when fluorescent signal became clear) under normal conditions on *E*. *coli* OP50 or HT115 RNAi control strains at 20℃. For strains with the *glp*‐*1* mutation, eggs were kept at 20℃ for 2–6 h then grown to Day 1 at 25℃. Day 1 adults were transferred to PA14 plates or OP50 control plates and maintained 25℃ for 24 h before imaging and quantification. Each strain was washed into the COPAS sample cup with ~5 ml deionized water for measurement of individual worms. Intensity of green fluorescence of each animal was normalized to the axial length measured (i.e., GFP fluorescence divided by time‐of‐flight). Statistical significance was determined using one‐way nonparametric ANOVA with Dunn’s post hoc test (Graphpad Prism). Representative whole‐body images of transgenic worms were taken using 10 mM Sodium Azide for immobilization and imaged at 20× magnification using a Leica DM5500B compound scope with LAS X software (Leica).

### 4.9 NHR‐49:GFP nuclear localization

Worms were grown to the L4 on OP50 and transferred to control *E*. *coli* OP50 or PA14 plates as described above. Following 16 h of exposure, animals were immobilized and mounted on agar pads with 20 mM Levamisole and imaged using a Leica DM5500B compound scope. Image acquisition and analysis was performed using LAS X software (Leica). For each of the two trials, an average of two to four of the first six anterior intestinal cells was assessed for GFP localization from ≥10 worms. For each intestinal cell, the size normalized nucleus‐to‐cytoplasm GFP intensity ratio was calculated using the Fiji (ImageJ) software by selecting the nuclear or cytoplasmic area and measuring corrected total cell fluorescence (CTCF) to obtain the nuclear CTCF/cytoplasmic CTCF ratio per cell. An unpaired *t* test with Welch’s correction was used to determine statistical significance.

## CONFLICT OF INTEREST

The authors declare no conflict of interest.

## AUTHOR CONTRIBUTIONS

AG conceived the project and designed the experiments; NN, FG, RR, JL, NB, and AG performed the experiments; AG and NN wrote the manuscript with input from the other authors.

## Supporting information

Fig S1

Fig S2

Fig S3

Fig S4

Fig S5

Fig S6

Table S1

Table S2‐S11

## Data Availability

The experimental data, including RNAseq data, are available in the supplementary materials of this publication. Materials and protocols are detailed in the methods section and further questions can be sent to the corresponding author. The raw RNAseq data has been uploaded to GEO and is available with accession series GSE158729. this information has been added to the methods section.
