# Supplementary material for: Cell nonautonomous roles of NHR‐49 in promoting longevity and innate immunity
Source: Aging Cell. 2021 Jun 22;20(7):e13413. doi: 10.1111/acel.13413 (PMC8282243; doi:10.1111/acel.13413)
Supplement: Supplementary file 1 — Fig S1 [file ACEL-20-e13413-s004.pdf]

(S1a) Strains compared by RNA-Seq in present study

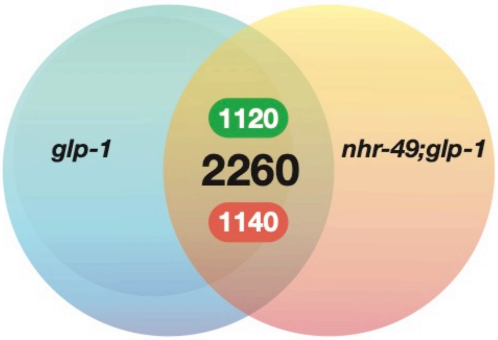

(S1b) Comparison of NHR-49 targets with DAF-16 and TCER-1 targets in *glp-1* mutants

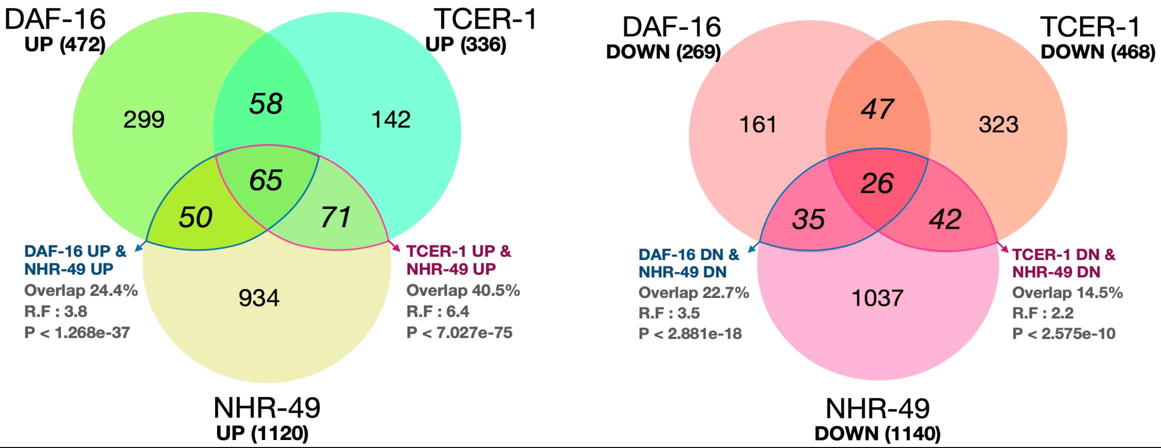

(S1c) NHR-49 UP genes (this study) with previously-identified roles in *glp-1* longevity *(Amrit et al., PLoS Gen. 2016)*

Wormbase Gene ID	Public Name	Overlapping Group	Avg. % Suppression of Lifespan
WBGene00000966	*dhs-2*	DAF-16 & TCER-1 UP	-14
WBGene00000981	*dhs-18*	DAF-16 & TCER-1 UP	-12
WBGene00001387	*far-3*	DAF-16 & TCER-1 UP	-12
WBGene00001500	*ftn-1*	DAF-16 & TCER-1 UP	-18
WBGene00001777	*gst-29*	DAF-16 & TCER-1 UP	-15
WBGene00004818	*skr-12*	DAF-16 & TCER-1 UP	-22
WBGene00004994	*spp-9*	DAF-16 & TCER-1 UP	-12
WBGene00004997	*spp-12*	DAF-16 & TCER-1 UP	-14
WBGene00007140	*cyp-29A4*	DAF-16 & TCER-1 UP	-15
WBGene00010706	*cyp-14A2*	DAF-16 & TCER-1 UP	-17
WBGene00011362	*cest-1.1*	DAF-16 & TCER-1 UP	-19
WBGene00013904	*ugt-6*	DAF-16 & TCER-1 UP	-21
WBGene00017390	F12A10.1	DAF-16 & TCER-1 UP	-11
WBGene00020413	T10E9.3	DAF-16 & TCER-1 UP	-22
WBGene00020909	W01A11.1	DAF-16 & TCER-1 UP	-17
WBGene00000975	*dhs-12*	DAF-16 UP	-17
WBGene00003473	*mtl-1*	DAF-16 UP	-22
WBGene00012404	Y6G8.2	DAF-16 UP	-18
WBGene00014030	*glb-1*	DAF-16 UP	-15
WBGene00021491	*comt-4*	DAF-16 UP	-11
WBGene00000982	*dhs-19*	TCER-1 UP	-22
WBGene00000986	*dhs-23*	TCER-1 UP	-20
WBGene00000988	*dhs-25*	TCER-1 UP	-21
WBGene00007362	*cyp-35C1*	TCER-1 UP	-17
WBGene00007848	*cytb-5.1*	TCER-1 UP	-21
WBGene00007963	*cyp-25A1*	TCER-1 UP	-10
WBGene00008499	*cyp-37A1*	TCER-1 UP	-22
WBGene00010659	K08D8.5	TCER-1 UP	-15
WBGene00011830	*cyp-29A2*	TCER-1 UP	-10
WBGene00013594	Y872A.2	TCER-1 UP	-15
WBGene00015400	*cyp-35A2*	TCER-1 UP	-16
WBGene00015692	*ugt-25*	TCER-1 UP	-14
WBGene00016786	*cyp-35A4*	TCER-1 UP	-20
WBGene00018958	*cest-27*	TCER-1 UP	-11
WBGene00019473	*cyp-35A5*	TCER-1 UP	-13
