# Supplementary figures and images for: Cell nonautonomous roles of NHR‐49 in promoting longevity and innate immunity
Source: Aging Cell. 2021 Jun 22;20(7):e13413. doi: 10.1111/acel.13413 (PMC8282243; doi:10.1111/acel.13413)

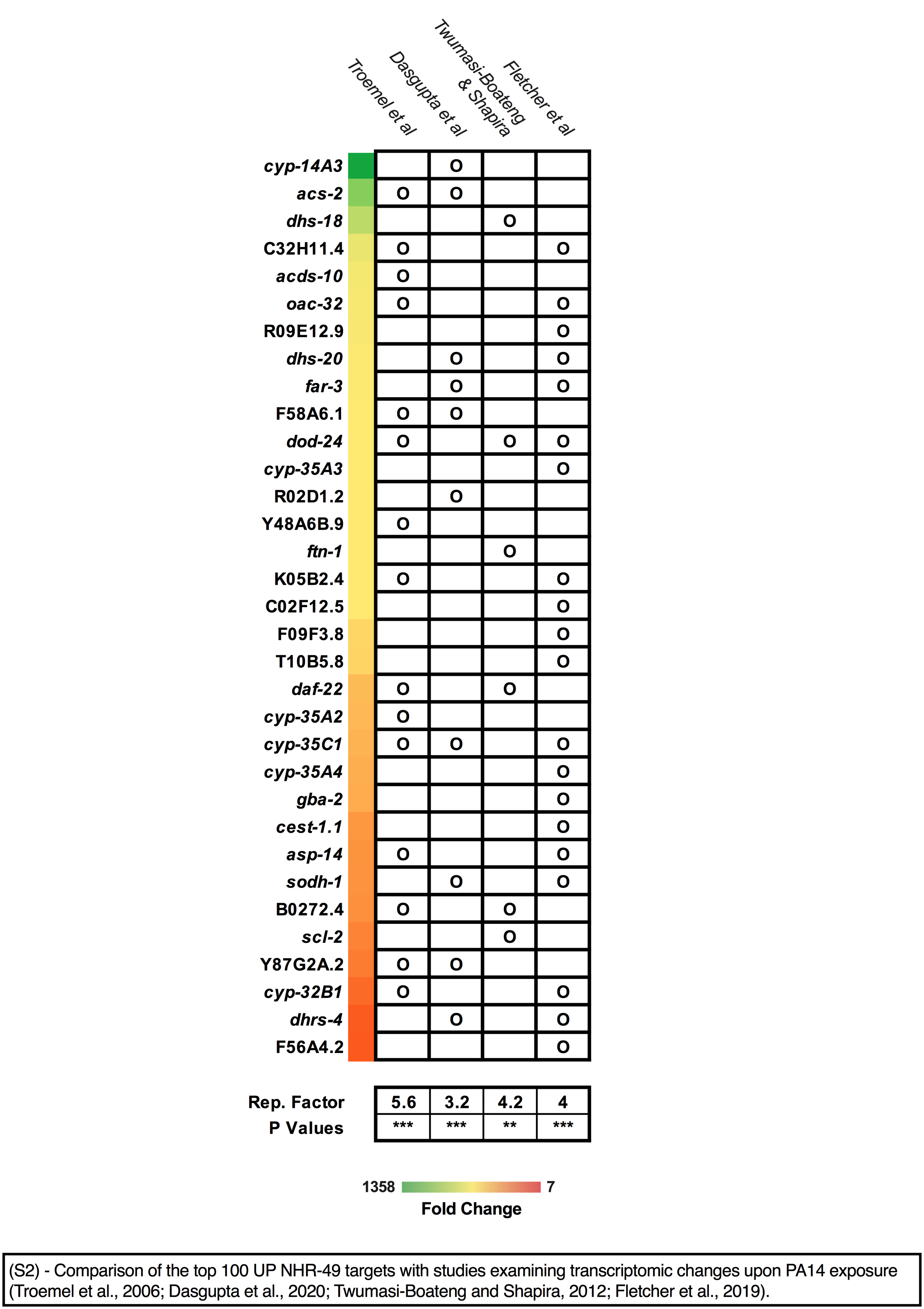

Supplement: Supplementary file 2 — Fig S2 [file ACEL-20-e13413-s003.tiff]

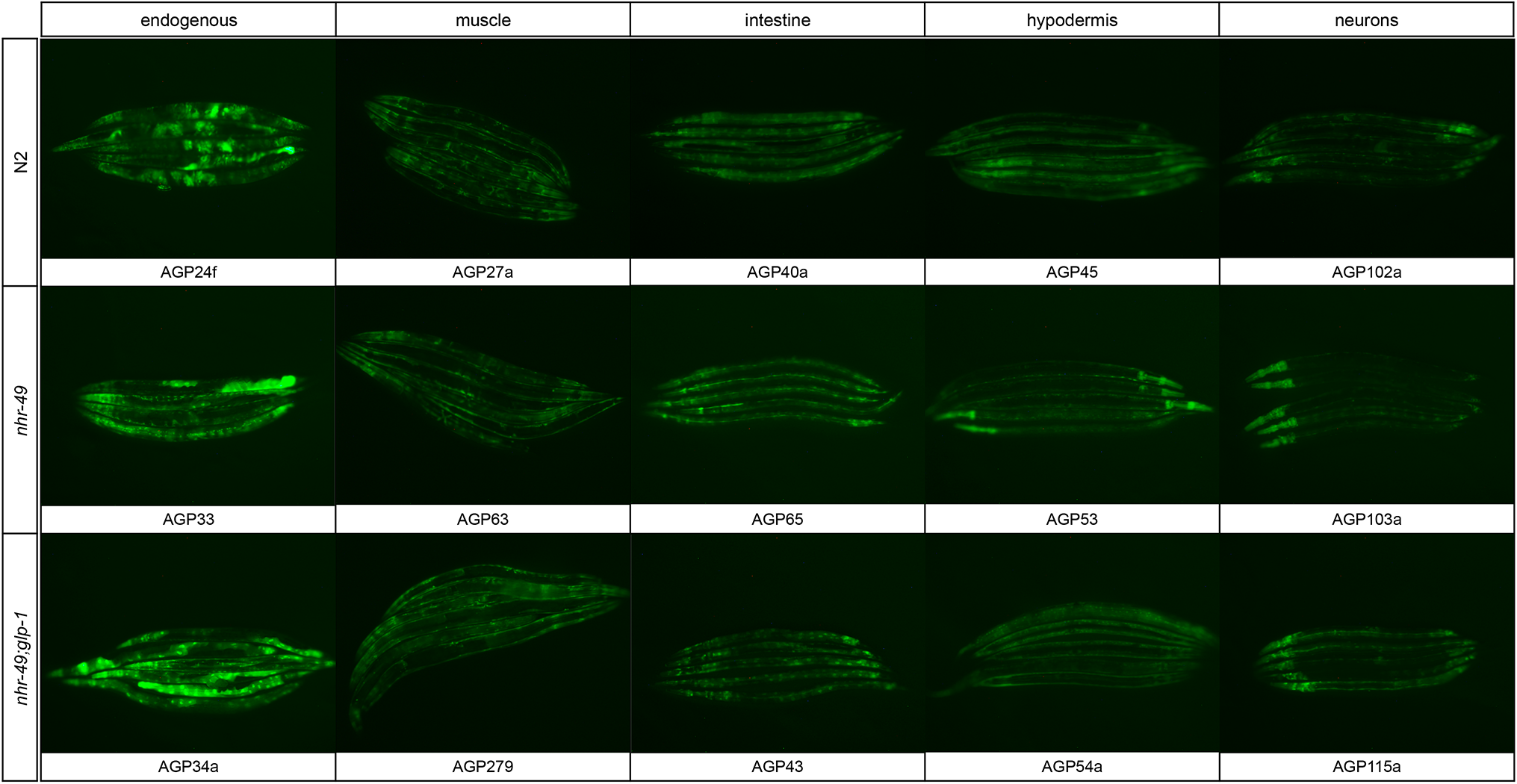

Supplement: Supplementary file 3 — Fig S3 [file ACEL-20-e13413-s001.tif]

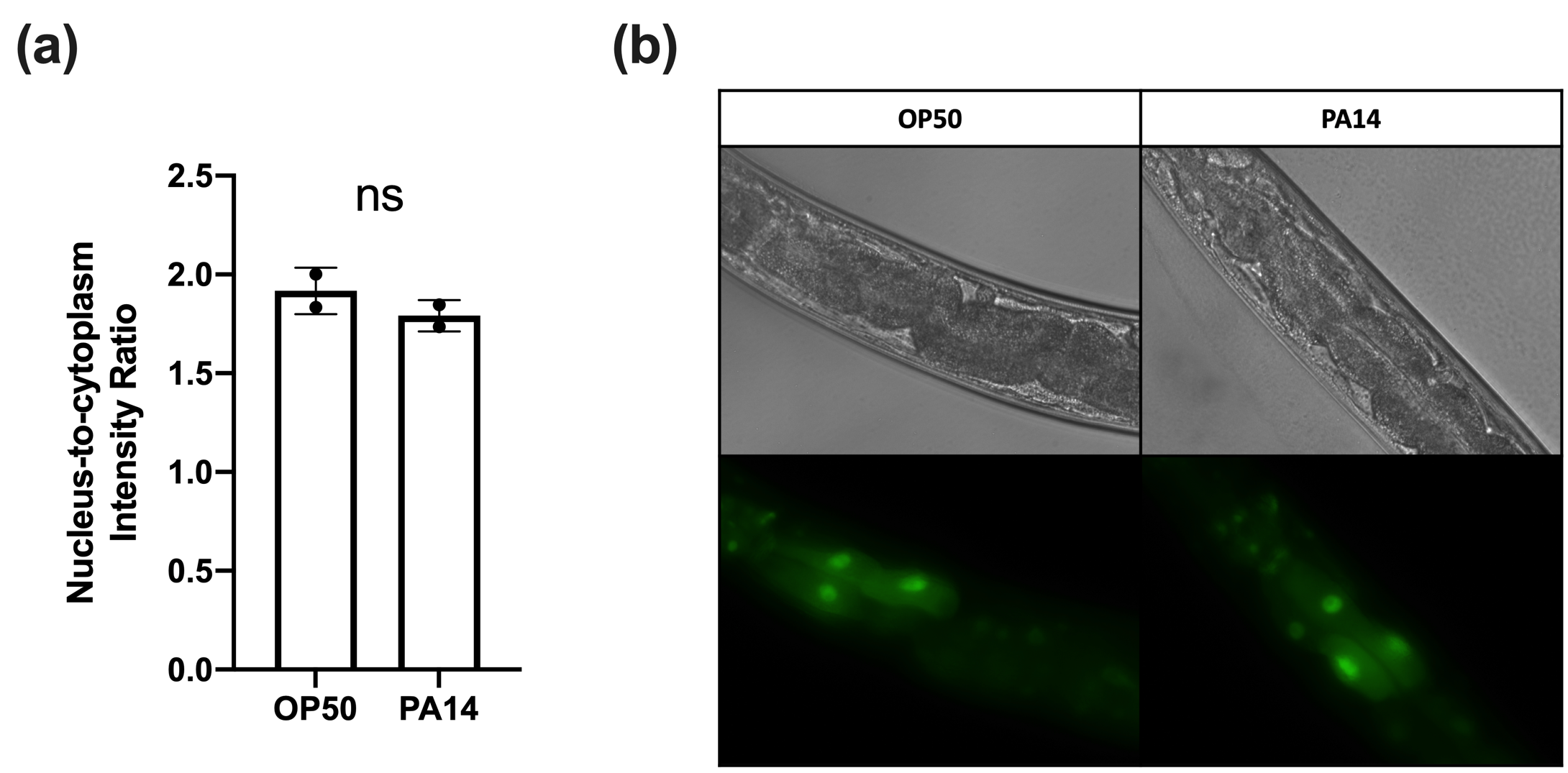

Supplement: Supplementary file 4 — Fig S4 [file ACEL-20-e13413-s002.tiff]

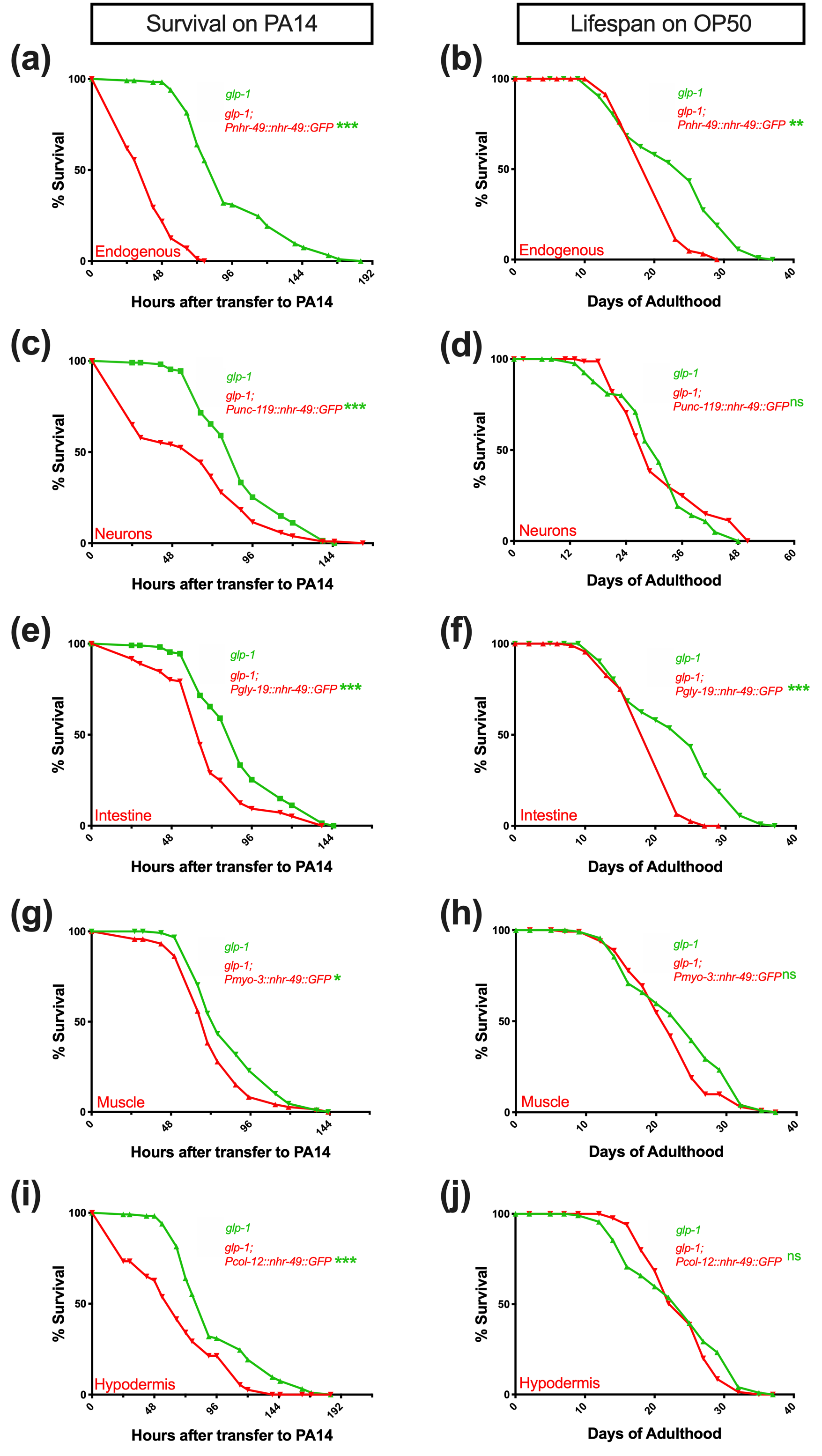

Supplement: Supplementary file 5 — Fig S5 [file ACEL-20-e13413-s007.tiff]

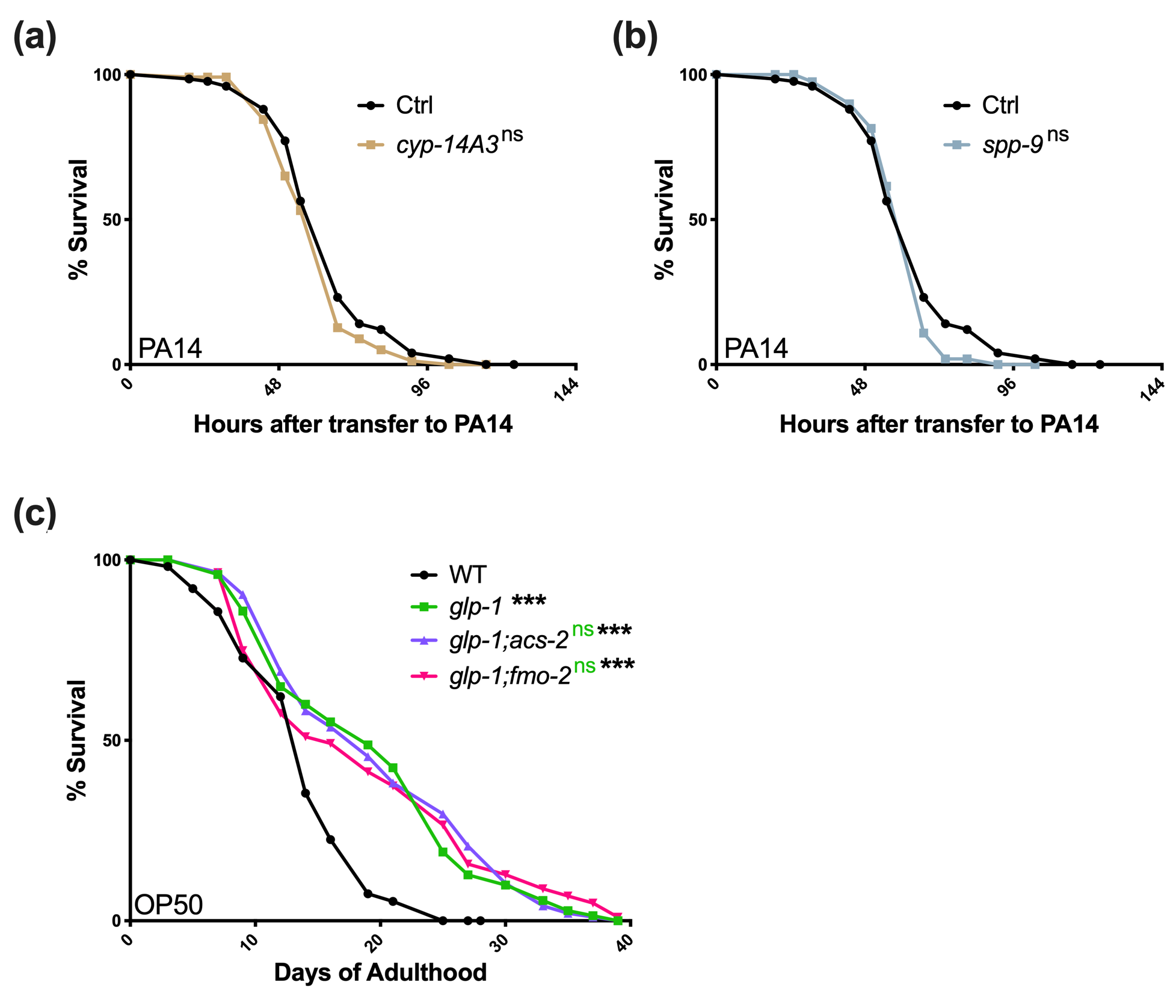

Supplement: Supplementary file 6 — Fig S6 [file ACEL-20-e13413-s006.tiff]
