# Supplementary material for: Cell nonautonomous roles of NHR‐49 in promoting longevity and innate immunity
Source: Aging Cell. 2021 Jun 22;20(7):e13413. doi: 10.1111/acel.13413 (PMC8282243; doi:10.1111/acel.13413)
Supplement: Supplementary file 8 — Table S2‐S11 [file ACEL-20-e13413-s005.pdf]

Table S2a: Impact of *nhr-49(et7)* on PA14 survival (A.1) and OP50 lifespan (A.2)

Genotype	Trial 1			Bonferroni P value
	n = obs/ total	Mean (h)	SE +/-	P (*vs* N2)
**Table S2a.1: SURVIVAL ON PA14**				
WT	126 / 145	54.32	1.09	
nhr-49(et7) gof	94 / 136	62.79	1.31	<0.0001
**Trial 2**				
WT	60 / 117	78.4	2.66	
nhr-49(et7) gof	79 / 110	74.68	3.67	0.4354
**Trial 3**				
WT	102 / 129	76.98	2.25	
nhr-49(et7) gof	73 / 118	76.09	2.57	1
**Trial 4#**				
WT	87 / 150	77.36	1.79	
nhr-49(et7) gof	60 / 130	89.35	2.22	<0.0001
**Trial 5~**				
WT	94 / 120	75.16	2.74	
nhr-49(et7) gof	94 / 140	76.77	2.3	1
**Trial 6$**				
WT	78 / 106	67.46	2.15	
nhr-49(et7) gof	93 / 125	77.88	1.85	0.0002

Table S2a.2: SURVIVAL ON OP50

Genotype	Trial 1◇			Bonferroni P value
	n = obs/ total	Mean (h)	SE +/-	P (*vs* N2)
WT	99 / 120	15.61	0.46	
nhr-49(et7) gof	78 / 118	16.75	0.54	0.4299
**Trial 2†**				
WT	103 / 136	16.49	0.44	
nhr-49(et7) gof	86 / 117	13.46	0.66	0.0422

Special characters #, ~, $, ◇, † on trial headings denote the trial was performed alongside similarly-labeled experiments in Table S6A.
Underlined Trials in S2a and S2b shown in Figs. 2b and 2c, respectively.

Table S2b: Impact of Fenofibrate on survival upon PA14 exposure

SURVIVAL ON PA14

Genotype	Treatment	Trial 1			Bonferroni P-value	
		n = obs/ total	Mean (h)	SE +/-	P (*vs* WT Control)	P (*vs* FF Control)
WT	Control	103 / 129	56.81	1.08		
	Fenofibrate	93 / 135	62.1	1.26	0.0041	0.0041
nhr-49	Control	107 / 134	47.69	0.98	<0.0001	
	Fenofibrate	104 / 134	45.33	0.89	<0.0001	0.072
**Trial 2**						
WT	Control	82 / 131	61.69	0.99		
	Fenofibrate	78 / 137	65.92	1.78	< 0.0001	< 0.0001
nhr-49	Control	83 / 130	41.33	0.71	< 0.0001	
	Fenofibrate	94 / 131	41.36	0.8	< 0.0001	0.0004
**Trial 3**						
WT	Control	103 / 150	59.21	1.09		
	Fenofibrate	104 / 150	63.16	1.43	0.0187	0.0187
nhr-49	Control	62 / 119	44.82	0.76	< 0.0001	
	Fenofibrate	110 / 149	40.99	0.83	< 0.0001	1
**Trial 4**						
WT	Control	131 / 160	66	1.19		
	Fenofibrate	148 / 160	71.86	1.11	0.0006	0.0006
nhr-49	Control	126 / 160	47.92	0.7	< 0.0001	
	Fenofibrate	152 / 161	47.44	0.71	< 0.0001	1
**Trial 5**						
WT	Control	108 / 150	66.75	1.37		
	Fenofibrate	90 / 145	64.73	1.32	1	1
nhr-49	Control	116 / 122	50.49	0.99	< 0.0001	
	Fenofibrate	110 / 120	51.3	0.95	< 0.0001	1
**Trial 6**						
WT	Control	103 / 120	78.28	1.73		
	Fenofibrate	84 / 120	70.11	1.42	0.0017	0.0017
nhr-49	Control	150 / 159	51.94	0.72	< 0.0001	
	Fenofibrate	148 / 160	52.91	0.77	< 0.0001	1
**Trial 7**						
WT	Control	95 / 146	61.37	1.46		
	Fenofibrate	91 / 139	63.24	1.66	0.041	0.041
nhr-49	Control	102 / 146	46.38	0.88	< 0.0001	
	Fenofibrate	123 / 151	48.08	0.65	< 0.0001	< 0.0001
**Trial 8**						
WT	Control	53/150	70.09	2.47		
	Fenofibrate	65/150	61.58	2.17	0.0066	0.0066
nhr-49	Control	127/150	46.82	0.76	< 0.0001	
	Fenofibrate	108/150	41.86	0.49	< 0.0001	< 0.0001

Table S3a: Impact of endogenous promoter-driven NHR-49 expression on survival of *nhr-49;glp-1* mutants on PA14 (a.1) or OP50 (a.2)

Table S3a.1: SURVIVAL ON PA14

Strain	Background/Genotype	Trial 1*			Bonferroni P-value		
		n = obs/ total	Mean (h)	SE +/-	P (*vs* N2)	P (*vs nhr-49; glp-1*)	P (*vs glp-1*)
WT	Wild type	100 / 122	59.17	1.14			
AGP22	*nhr-49;glp-1*	111 / 121	56.25	1.42	0.7488		
CF1903	*glp-1*	117 / 124	80.71	1.62	<0.0001	<0.0001	
AGP34	*nhr-49;glp-1 glmEx5 [Pnhr-49::nhr-49::GFP + Pmyo-2::mCh]*	79 / 113	43.77	1.36	<0.0001	<0.0001	<0.0001
**Trial 2#**							
WT	Wild type	68/120	72.12	2.14			
AGP22	*nhr-49;glp-1*	108/120	57.92	1.35	<0.0001		
CF1903	*glp-1*	105/120	82.88	2.08	0.0042	<0.0001	
AGP34	*nhr-49;glp-1 glmEx5 [Pnhr-49::nhr-49::GFP + Pmyo-2::mCh]*	95/120	43.33	1.74	<0.0001	<0.0001	<0.0001
**Trial 3^**							
WT	Wild type	67 / 114	75.62	1.71			
AGP22	*nhr-49;glp-1*	98 / 111	53.32	1.26	<0.0001		
CF1903	*glp-1*	104 / 108	89.65	2.83	0.0429	<0.0001	
AGP34	*nhr-49;glp-1 glmEx5 [Pnhr-49::nhr-49::GFP + Pmyo-2::mCh]*	69 / 78	35.59	1.37	<0.0001	<0.0001	<0.0001
**Trial 4$**							
WT	Wild type	88 / 128	92.05	1.99			
AGP22	*nhr-49;glp-1*	106 / 132	69.89	1.69	<0.0001		
CF1903	*glp-1*	101 / 131	106.42	2.74	0.0004	<0.0001	
AGP34	*nhr-49;glp-1 glmEx5 [Pnhr-49::nhr-49::GFP + Pmyo-2::mCh]*	80 / 111	54.13	2.67	<0.0001	<0.0001	<0.0001

Table S3a.2: SURVIVAL ON OP50

Strain	Background Genotype	Trial 1@			Bonferroni P-value		
		n = obs/ total	Mean (days)	SE +/-	P (*vs* N2)	P (*vs nhr-49;glp-1*)	P (*vs glp-1*)
AGP22	*nhr-49;glp-1*	72/72	11.27	0.26			
CF1903	*glp-1*	65/77	25.78	0.98		<0.0001	
AGP34	*nhr-49;glp-1 glmEx5 [Pnhr-49::nhr-49::GFP + Pmyo-2::mCh]*	14/26	24.6	1.39		<0.0001	1
**Trial 2&**							
WT	Wild type	52/71	16.26	0.91			
AGP22	*nhr-49;glp-1*	55/72	11.28	0.26	0.0002		
CF1903	*glp-1*	51/70	24.43	1.08	<0.0001	<0.0001	
AGP34	*nhr-49;glp-1 glmEx5 [Pnhr-49::nhr-49::GFP + Pmyo-2::mCh]*	59/60	23.72	1.32	<0.0001	<0.0001	1
**Trial 3~**							
AGP22	*nhr-49;glp-1*	125 / 142	9.96	0.07			
CF1903	*glp-1*	100 / 107	18.49	0.53		<0.0001	
AGP34	*nhr-49;glp-1 glmEx5 [Pnhr-49::nhr-49::GFP + Pmyo-2::mCh]*	102 / 124	15.61	0.58		<0.0001	0.0046

*, #, ^, $, & and ~ on different tabs/sub-sections of Table S3 denote trials performed at the same time

Underlined Trials shown in Fig. 3

Table S3b: Impact of neuronal (*unc-119*) promoter-driven NHR-49 expression on survival of *nhr-49;glp-1* mutants on PA14 (b.1) or OP50 (b.2)

Table S3b. 1: SURVIVAL ON PA14

Strain	Background/Genotype	Trial 1*			Bonferroni P-value		
		n = obs/ total	Mean (h)	SE +/-	P (*vs* N2)	P (*vs nhr-49;glp-1*)	P (*vs glp-1*)
WT	Wild type	100 / 122	59.17	1.14			
AGP22	*nhr-49;glp-1*	111 / 121	56.25	1.42	0.7488		
CF1903	*glp-1*	117 / 124	80.71	1.62	<0.0001	<0.0001	
AGP115	*nhr-49;glp-1 glmEx29 [Punc-119:: nhr-49::GFP + Pmyo-2::mCh]*	107 / 120	74.76	2.92	<0.0001	<0.0001	1
**Trial 2#**							
WT	Wild type	68/120	72.12	2.14			
AGP22	*nhr-49;glp-1*	108/120	57.92	1.35	<0.0001		
CF1903	*glp-1*	105/120	82.88	2.08	0.0042	<0.0001	
AGP115	*nhr-49;glp-1 glmEx29 [Punc-119:: nhr-49::GFP + Pmyo-2::mCh]*	104/120	77.91	3.28	0.1476	<0.0001	1
**Trial 3^**							
WT	Wild type	67 / 114	75.62	1.71			
AGP22	*nhr-49;glp-1*	98 / 111	53.32	1.26	<0.0001		
CF1903	*glp-1*	104 / 108	89.65	2.83	0.0429	<0.0001	
AGP115	*nhr-49;glp-1 glmEx29 [Punc-119:: nhr-49::GFP + Pmyo-2::mCh]*	90 / 102	92.09	3.49	0.0028	<0.0001	1

Table Sb.2: SURVIVAL ON OP50

Strain	Background Genotype	Trial 1&			Bonferroni P-value		
		n = obs/ total	Mean (days)	SE +/-	P (*vs* N2)	P (*vs nhr-49;glp-1*)	P (*vs glp-1*)
WT	Wild type	52/71	16.26	0.91			
AGP22	*nhr-49;glp-1*	55/72	11.28	0.26	0.0002		
CF1903	*glp-1*	51/70	24.43	1.08	<0.0001	<0.0001	
AGP115	*nhr-49;glp-1 glmEx29 [Punc-119:: nhr-49::GFP + Pmyo-2::mCh]*	66/71	24.41	1.04	<0.0001	<0.0001	1
**Trial 2**							
WT	Wild type	71 / 100	23.8	0.58			
AGP22	*nhr-49;glp-1*	110 / 145	11.38	0.19	<0.0001		
CF1903	*glp-1*	83 / 84	22.58	1.06	1	<0.0001	
AGP115	*nhr-49;glp-1 glmEx29 [Punc-119:: nhr-49::GFP + Pmyo-2::mCh]*	67 / 92	23.97	0.45	1	<0.0001	1
**Trial 3**							
WT	Wild type	71 / 82	17.99	0.58			
AGP22	*nhr-49;glp-1*	63 / 65	11.11	0.33	<0.0001		
CF1903	*glp-1*	109 / 121	24.25	0.91	<0.0001	<0.0001	
AGP115	*nhr-49;glp-1 glmEx29 [Punc-119:: nhr-49::GFP + Pmyo-2::mCh]*	83 / 103	24.9	0.55	<0.0001	<0.0001	1

*, #, ^, $, & and ~ on different tabs/sub-sections of Table S3 denote trials performed at the same time

Underlined Trials shown in Fig. 3

Table S3c: Impact of intestinal (*gly-19*) promoter-driven NHR-49 expression on survival of *nhr-49;glp-1* mutants on PA14 (c.1) and OP50 (c.2)

Table S3c. 1: SURVIVAL ON PA14

Strain	Background/Genotype	Trial 1*			Bonferroni P-value		
		n = obs/ total	Mean (h)	SE +/-	P (*vs* N2)	P (*vs nhr-49;glp-1*)	P (*vs glp-1*)
WT	Wildtype	100 / 122	59.17	1.14			
AGP22	*nhr-49;glp-1*	111 / 121	56.25	1.42	0.7488		
CF1903	*glp-1*	117 / 124	80.71	1.62	<0.0001	<0.0001	
AGP43	*nhr-49;glp-1 glmEx10 [Pgly-19::nhr-49::GFP + Pmyo-2::mCherry]*	68 / 79	47.82	2.57	0.0026	0.0693	<0.0001
**Trial 2$**							
WT	Wildtype	88 / 128	92.05	1.99			
AGP22	*nhr-49;glp-1*	106 / 132	69.89	1.69	<0.0001		
CF1903	*glp-1(e2144)*	101 / 131	106.42	2.74	0.0004	<0.0001	
AGP43	*nhr-49;glp-1 glmEx10 [Pgly-19::nhr-49::GFP + Pmyo-2::mCherry]*	86 / 140	77.36	3.45	0.0086	0.0726	<0.0001
**Trial 3**							
WT	Wildtype	94 / 135	89.9	2.17			
AGP22	*nhr-49;glp-1*	124 / 139	61.35	1.3	<0.0001		
CF1903	*glp-1(e2144)*	94 / 139	93.57	2.96	1	<0.0001	
AGP43	*nhr-49;glp-1 glmEx10 [Pgly-19::nhr-49::GFP + Pmyo-2::mCherry]*	93 / 140	61.68	2.39	<0.0001	1	<0.0001

Table S3c.2: SURVIVAL ON OP50

Strain	Background Genotype	Trial 1@			Bonferroni P-value		
		n = obs/ total	Mean (days)	SE +/-	P (*vs* N2)	P (*vs nhr-49;glp-1*)	P (*vs glp-1*)
AGP22	*nhr-49;glp-1*	72/72	11.27	0.26			
CF1903	*glp-1(e2144)*	65/77	25.78	0.98		<0.0001	
AGP43	*nhr-49;glp-1 glmEx10 [Pgly-19::nhr-49::GFP + Pmyo-2::mCherry]*	41/63	19.79	0.95		<0.0001	0.0002
**Trial 2~**							
AGP22	*nhr-49;glp-1*	125 / 142	9.96	0.07			
CF1903	*glp-1(e2144)*	100 / 107	18.49	0.53		<0.0001	
AGP43	*nhr-49;glp-1 glmEx10 [Pgly-19::nhr-49::GFP + Pmyo-2::mCherry]*	118 / 137	16.55	0.2		<0.0001	0.0011

*, #, ^, $, & and ~ on different tabs/sub-sections of Table S3 denote trials performed at the same time
Underlined Trials shown in Fig. 3

Table S3d: Impact of muscle (*myo-3*) promoter-driven NHR-49 expression on survival of *nhr-49;glp-1* mutants on PA14 (d.1) and OP50 (d.2)

Table S3d. 1: SURVIVAL ON PA14							
**Strain**	**Background/Genotype**	**Trial 1***			**Bonferroni P-value**		
		**n = obs/ total**	**Mean (h)**	**SE +/-**	**P (*vs* N2)**	**P (*vs nhr-49;glp-1*)**	**P (*vs glp-1*)**
WT	Wildtype	100 / 122	59.17	1.14			
AGP22	*nhr-49;glp-1*	111 / 121	56.25	1.42	0.7488		
CF1903	*glp-1*	117 / 124	80.71	1.62	<0.0001	<0.0001	
AGP279	*nhr-49;glp-1 glmEx7 [Pmyo-3::nhr-49::GFP + Pmyo-2::mCherry]*	109 / 115	52.44	1.67	0.3334	1	<0.0001
**Trial 2#**							
WT	Wildtype	68/120	72.12	2.14			
AGP22	*nhr-49;glp-1*	108/120	57.92	1.35	<0.0001		
CF1903	*glp-1*	105/120	82.88	2.08	0.0042	<0.0001	
AGP279	*nhr-49;glp-1 glmEx7 [Pmyo-3::nhr-49::GFP + Pmyo-2::mCherry]*	108/120	62.52	2.29	0.1479	<0.0001	<0.0001
**Trial 3^**							
WT	Wildtype	67 / 114	75.62	1.71			
AGP22	*nhr-49;glp-1*	98 / 111	53.32	1.26	<0.0001		
CF1903	*glp-1*	104 / 108	89.65	2.83	0.0429	<0.0001	
AGP279	*nhr-49;glp-1 glmEx7 [Pmyo-3::nhr-49::GFP + Pmyo-2::mCherry]*	105 / 115	63.53	1.76	<0.0001	<0.0001	<0.0001

Table S3d.2: SURVIVAL ON OP50							
**Strain**	**Background Genotype**	**Trial 1@**			**Bonferroni P-value**		
		**n = obs/ total**	**Mean (days)**	**SE +/-**	**P (*vs* N2)**	**P (*vs nhr-49;glp-1*)**	**P (*vs glp-1*)**
AGP22	*nhr-49;glp-1*	72/72	11.27	0.26			
CF1903	*glp-1*	65/77	25.78	0.98		<0.0001	
AGP279	*nhr-49;glp-1 glmEx7 [Pmyo-3::nhr-49::GFP + Pmyo-2::mCherry]*	34/59	21.4	1.02		<0.0001	0.0113
**Trial 2~**							
AGP22	*nhr-49;glp-1*	125 / 142	9.96	0.07			
CF1903	*glp-1*	100 / 107	18.49	0.53		<0.0001	
AGP279	*nhr-49;glp-1 glmEx7 [Pmyo-3::nhr-49::GFP + Pmyo-2::mCherry]*	109 / 116	17.71	0.33		<0.0001	1

*, #, ^, $, & and ~ on different tabs/sub-sections of Table S3 denote trials performed at the same time
Underlined Trials shown in Fig. 3

Table S3e: Impact of hypodermal (*col-12*) promoter-driven NHR-49 expression on survival of *nhr-49;glp-1* mutants on PA14 (e.1) and OP50 (e.2)

Strain	Background/Genotype	n = obs/ total	Mean (h)	SE +/-	P (*vs* N2)	P (*vs nhr-49;glp-1*)	P (*vs glp-1*)
**Table S3e.1: SURVIVAL ON PA14**							
		**Trial 1***			**Bonferroni P-value**		
WT	Wildtype	100 / 122	59.17	1.14			
AGP22	*nhr-49;glp-1*	111 / 121	56.25	1.42	0.7488		
CF1903	*glp-1*	117 / 124	80.71	1.62	<0.0001	<0.0001	
AGP54	*nhr-49;glp-1 glmEx11 [Pcol-12::nhr-49::GFP + Pmyo-2::mCherry]*	97 / 115	57.83	2.42	1	1	<0.0001
**Trial 2#**							
WT	Wildtype	68/120	72.12	2.14			
AGP22	*nhr-49;glp-1*	108/120	57.92	1.35	<0.0001		
CF1903	*glp-1*	105/120	82.88	2.08	0.0042	<0.0001	
AGP54	*nhr-49;glp-1 glmEx11 [Pcol-12::nhr-49::GFP + Pmyo-2::mCherry]*	96/120	56.06	2.53	0.0004	1	<0.0001
**Trial 3^**							
WT	Wildtype	67 / 114	75.62	1.71			
AGP22	*nhr-49;glp-1*	98 / 111	53.32	1.26	<0.0001		
CF1903	*glp-1*	104 / 108	89.65	2.83	0.0429	<0.0001	
AGP54	*nhr-49;glp-1 glmEx11 [Pcol-12::nhr-49::GFP + Pmyo-2::mCherry]*	89 / 104	66.00	2.7	0.0508	<0.0001	<0.0001

Strain	Background Genotype	n = obs/ total	Mean (days)	SE +/-	P (*vs* N2)	P (*vs nhr-49;glp-1*)	P (*vs glp-1*)
**Table S3e.2: SURVIVAL ON OP50**							
		**Trial 1@**			**Bonferroni P-value**		
AGP22	*nhr-49;glp-1*	72/72	11.27	0.26			
CF1903	*glp-1*	65/77	25.78	0.98		<0.0001	
AGP54	*nhr-49;glp-1 glmEx11 [Pcol-12::nhr-49::GFP + Pmyo-2::mCherry]*	28/42	21.06	1.36		<0.0001	0.0593
**Trial 2~**							
AGP22	*nhr-49;glp-1*	125 / 142	9.96	0.07			
CF1903	*glp-1*	100 / 107	18.49	0.53		<0.0001	
AGP54	*nhr-49;glp-1 glmEx11 [Pcol-12::nhr-49::GFP + Pmyo-2::mCherry]*	118 / 136	16.61	0.13		<0.0001	0.0009

*, #, ^, $, & and ~ on different tabs/sub-sections of Table S3 denote trials performed at the same time
Underlined Trials shown in Fig. 3

Table S4a: Impact of endogenous promoter-driven NHR-49 expression on survival of *nhr-49* mutants on PA14 (a.1) and OP50 (a.2)

Table S4a.1: SURVIVAL ON PA14

Strain	Background/Genotype	Trial 1*			Bonferroni P-value	
		n = obs/ total	Mean (h)	SE +/-	P (*vs* N2)	P (*vs nhr-49*)
WT	Wildtype	125 / 155	67.25	1.86		
AGP12a	*nhr-49*	145 / 167	51.89	1.41	<0.0001	
AGP33	*nhr-49 glmEx5 [Pnhr-49::nhr-49::GFP + Pmyo-2::mCh]*	115 / 131	46.68	2.12	<0.0001	0.506
**Trial 2#**						
WT	Wildtype	93/150	68.58	2.05		
AGP12a	*nhr-49*	126/150	64.63	1.81	0.6022	
AGP33	*nhr-49 glmEx5 [Pnhr-49::nhr-49::GFP + Pmyo-2::mCh]*	87/100	74.96	3.35	0.7647	0.0101
**Trial 3^**						
WT	Wildtype	63/125	78.24	2.57		
AGP12a	*nhr-49*	99/127	58	1.44	<0.0001	
AGP33	*nhr-49 glmEx5 [Pnhr-49::nhr-49::GFP + Pmyo-2::mCh]*	87/127	94.98	3.08	0.002	<0.0001
**Trial 4$**						
WT	Wildtype	55/90	84.82	2.63		
AGP12a	*nhr-49*	102/113	60.24	1.52	0.0001	
AGP33	*nhr-49 glmEx5 [Pnhr-49::nhr-49::GFP + Pmyo-2::mCh]*	100/125	84.35	2.17	1	0.0001

Table S4a.2:SURVIVAL ON OP50

Strain	Background/Genotype	Trial 1@			Bonferroni P-value	
		n = obs/ total	Mean (h)	SE +/-	P (*vs* N2)	P (*vs nhr-49*)
WT	Wildtype	67/73	16.67	0.65		
AGP12a	*nhr-49*	33/77	14.39	0.69	0.1514	
AGP33	*nhr-49 glmEx5 [Pnhr-49::nhr-49::GFP + Pmyo-2::mCh]*	56/69	18.99	0.71	0.1646	0.0001
**Trial 2&**						
WT	Wildtype	89 / 122	15.42	0.49		
AGP12a	*nhr-49*	110 / 119	12.5	0.36	<0.0001	
AGP33	*nhr-49 glmEx5 [Pnhr-49::nhr-49::GFP + Pmyo-2::mCh]*	97 / 112	18	0.57	0.0424	<0.0001

*, #, ^, $, & on different tabs/sub-sections of Table S4 denote trials performed at the same time
Underlined Trials shown in Fig.4

Table S4b: Impact of neuronal (*unc-119*) promoter-driven NHR-49 expression on survival of *nhr-49* mutants on PA14 (b.1) and OP50 (b.2)

Table S4b. 1: SURVIVAL ON PA14

Strain	Background/Genotype	Trial 1*			Bonferroni P-value	
		n = obs/ total	Mean (h)	SE +/-	P (*vs* N2)	P (*vs* *nhr-49*)
WT	Wildtype	125 / 155	67.25	1.86		
AGP12a	*nhr-49*	145 / 167	51.89	1.41	<0.0001	
AGP103a	*nhr-49 glmEx20 [Punc-119:: nhr-49::GFP + Pmyo-2::mCh]*	113 / 124	57.87	2.82	0.9996	0.0017
**Trial 2#**						
WT	Wildtype	93/150	68.58	2.05		
AGP12a	*nhr-49*	126/150	64.63	1.81	0.6022	
AGP103a	*nhr-49 glmEx20 [Punc-119:: nhr-49::GFP + Pmyo-2::mCh]*	86/130	77.42	3.34	<0.0001	0.0002
**Trial 3^**						
WT	Wildtype	63/125	78.24	2.57		
AGP12a	*nhr-49*	99/127	58	1.44	<0.0001	
AGP103a	*nhr-49 glmEx20 [Punc-119:: nhr-49::GFP + Pmyo-2::mCh]*	96/125	81.54	3.19	0.7738	<0.0001
**Trial 4$**						
WT	Wildtype	55/90	84.82	2.63		
AGP12a	*nhr-49*	102/113	60.24	1.52	0.0001	
AGP103a	*nhr-49 glmEx20 [Punc-119:: nhr-49::GFP + Pmyo-2::mCh]*	95/110	84.44	2.73	1	0.0001

Table S4b.2: SURVIVAL ON OP50

Strain	Background/Genotype	Trial 1@			Bonferroni P-value	
		n = obs/ total	Mean (h)	SE +/-	P (*vs* N2)	P (*vs* *nhr-49*)
WT	Wildtype	67/73	16.67	0.65		
AGP12a	*nhr-49*	33/77	14.39	0.69	0.1514	
AGP103a	*nhr-49 glmEx20 [Punc-119:: nhr-49::GFP + Pmyo-2::mCh]*	48/57	14.58	0.63	0.1326	1
**Trial 2**						
WT	Wildtype	74 / 91	24.2	0.55		
AGP12a	*nhr-49*	70 / 75	14.29	0.22	<0.0001	
AGP103a	*nhr-49 glmEx20 [Punc-119:: nhr-49::GFP + Pmyo-2::mCh]*	87 / 95	13.98	0.2	<0.0001	1
**Trial 3**						
WT	Wildtype	71 / 100	23.8	0.58		
AGP12a	*nhr-49*	41 / 72	9.23	0.31	<0.0001	
AGP103a	*nhr-49 glmEx20 [Punc-119:: nhr-49::GFP + Pmyo-2::mCh]*	73 / 91	15.37	0.57	<0.0001	<0.0001
**Trial 4**						
WT	Wildtype	71 / 82	17.99	0.58		
AGP12a	*nhr-49*	63 / 65	11.11	0.33	<0.0001	
AGP103a	*nhr-49 glmEx20 [Punc-119:: nhr-49::GFP + Pmyo-2::mCh]*	33 / 76	22.23	0.71	0.0006	0.0002

*, #, ^, $, & on different tabs/sub-sections of Table S4 denote trials performed at the same time

Underlined Trials shown in Fig.4

**Table S4c: Impact of intestinal (*gly-19*) promoter-driven NHR-49 expression on survival of *nhr-49* mutants on PA14 (c.1) and OP50 (c.2)**						
**Table S4c. 1: SURVIVAL ON PA14**						
**Strain**	**Background/Genotype**	**Trial 1***			**Bonferroni P-value**	
		**n = obs/ total**	**Mean (h)**	**SE +/-**	**P (*vs* N2)**	**P (*vs nhr-49*)**
WT	Wildtype	125 / 155	67.25	1.86		
AGP12a	*nhr-49*	145 / 167	51.89	1.41	<0.0001	
AGP65	*nhr-49 glmEx9 [Pgly-19:: nhr-49 ::GFP + Pmyo-2::mCh]*	107 / 132	50.82	2.14	<0.0001	1
**Trial 2#**						
WT	Wildtype	93/150	68.58	2.05		
AGP12a	*nhr-49*	126/150	64.63	1.81	0.6022	
AGP65	*nhr-49 glmEx9 [Pgly-19:: nhr-49 ::GFP + Pmyo-2::mCh]*	89/150	73.78	2.92	1	0.0201
**Trial 3^**						
WT	Wildtype	63/125	78.24	2.57		
AGP12a	*nhr-49*	99/127	58	1.44	<0.0001	
AGP65	*nhr-49 glmEx9 [Pgly-19:: nhr-49 ::GFP + Pmyo-2::mCh]*	87/125	77.6	2.73	1	<0.0001
**Trial 4$**						
WT	Wildtype	55/90	84.82	2.63		
AGP12a	*nhr-49*	102/113	60.24	1.52	0.0001	
AGP65	*nhr-49 glmEx9 [Pgly-19:: nhr-49 ::GFP + Pmyo-2::mCh]*	100/116	75.5	2.37	0.2823	0.0001
**Table S4c.2: Survival on SURVIVAL ON OP50**						
**Strain**	**Background/Genotype**	**Trial 1@**			**Bonferroni P-value**	
		**n = obs/ total**	**Mean (h)**	**SE +/-**	**P (*vs* N2)**	**P (*vs nhr-49*)**
WT	Wildtype	67/73	16.67	0.65		
AGP12a	*nhr-49*	33/77	14.39	0.69	0.1514	
AGP65	*nhr-49 glmEx9 [Pgly-19:: nhr-49 ::GFP + Pmyo-2::mCh]*	47/56	15.85	0.71	1	1
**Trial 2&**						
WT	Wildtype	89 / 122	15.42	0.49		
AGP12a	*nhr-49*	110 / 119	12.5	0.36	<0.0001	
AGP65	*nhr-49 glmEx9 [Pgly-19:: nhr-49 ::GFP + Pmyo-2::mCh]*	97 / 118	15.25	0.4	1	<0.0001

*, #, ^, $, & on different tabs/sub-sections of Table S4 denote trials performed at the same time
Underlined Trials shown in Fig.4

Table S4d: Impact of muscle (*myo-3*) promoter-driven NHR-49 expression on survival of *nhr-49* mutants on PA14 (d.1) and OP50 (d.2)

Table S4d. 1: SURVIVAL ON PA14

Strain	Background/Genotype	Trial 1*			Bonferroni P-value	
		n = obs/ total	Mean (h)	SE +/-	P (*vs* N2)	P (*vs nhr-49*)
WT	Wildtype	125 / 155	67.25	1.86		
AGP12a	*nhr-49*	145 / 167	51.89	1.41	<0.0001	
AGP63	*nhr-49 glmEx8 [Pmyo-3::nhr-49 ::GFP + Pmyo-2::mCh]*	137 / 153	44.92	1.55	<0.0001	0.0819
**Trial 2#**						
WT	Wildtype	93/150	68.58	2.05		
AGP12a	*nhr-49*	126/150	64.63	1.81	0.6022	
AGP63	*nhr-49 glmEx8 [Pmyo-3::nhr-49 ::GFP + Pmyo-2::mCh]*	115/150	65.11	1.73	1	1
**Trial 3^**						
WT	Wildtype	63/125	78.24	2.57		
AGP12a	*nhr-49*	99/127	58	1.44	<0.0001	
AGP63	*nhr-49 glmEx8 [Pmyo-3::nhr-49 ::GFP + Pmyo-2::mCh]*	91/126	68.06	1.59	0.0124	<0.0001
**Trial 4$**						
WT	Wildtype	55/90	84.82	2.63		
AGP12a	*nhr-49*	102/113	60.24	1.52	0.0001	
AGP63	*nhr-49 glmEx8 [Pmyo-3::nhr-49 ::GFP + Pmyo-2::mCh]*	105/119	65.73	1.27	0.0001	0.0991

Table S4d.2: SURVIVAL ON OP50

Strain	Background/Genotype	Trial 1@			Bonferroni P-value	
		n = obs/ total	Mean (h)	SE +/-	P (*vs* N2)	P (*vs nhr-49*)
WT	Wildtype	67/73	16.67	0.65		
AGP12a	*nhr-49*	33/77	14.39	0.69	0.1514	
AGP63	*nhr-49 glmEx8 [Pmyo-3::nhr-49 ::GFP + Pmyo-2::mCh]*	73/75	11.84	0.54	0.0001	0.0893
**Trial 2&**						
WT	Wildtype	89 / 122	15.42	0.49		
AGP12a	*nhr-49*	110 / 119	12.5	0.36	<0.0001	
AGP63	*nhr-49 glmEx8 [Pmyo-3::nhr-49 ::GFP + Pmyo-2::mCh]*	75 / 80	12.01	0.71	0.0001	1

*, #, ^, $, & on different tabs/sub-sections of Table S4 denote trials performed at the same time
Underlined Trials shown in Fig.4

Table S4e: Impact of hypodermal (*col-12*) promoter-driven NHR-49 expression on survival of *nhr-49* mutants on PA14 (e.1) and OP50 (e.2)

Table S4e. 1: SURVIVAL ON PA14

Strain	Background/Genotype	Trial 1* n = obs/ total	Mean (h)	SE +/-	Bonferroni P-value P (*vs* N2)	P (*vs nhr 49*)
WT	Wildtype	125 / 155	67.25	1.86		
AGP12a	*nhr-49*	145 / 167	51.89	1.41	<0.0001	
AGP53	*nhr-49 glmEx11 [Pcol-12::nhr-49::GFP + Pmyo-2::mCh]*	138 / 150	30.21	1.77	<0.0001	<0.0001
		**Trial 2#**				
WT	Wildtype	93/150	68.58	2.05		
AGP12a	*nhr-49*	126/150	64.63	1.81	0.6022	
AGP63	*nhr-49 glmEx8 [Pmyo-3::nhr-49::GFP + Pmyo-2::mCh]*	90/128	48.55	2.56	<0.0001	0.0001
		**Trial 3^**				
WT	Wildtype	63/125	78.24	2.57		
AGP12a	*nhr-49*	99/127	58	1.44	<0.0001	
AGP53	*nhr-49 glmEx11 [Pcol-12::nhr-49::GFP + Pmyo-2::mCh]*	79/100	46.27	2.72	<0.0001	0.0415
		**Trial 4$**				
WT	Wildtype	55/90	84.82	2.63		
AGP12a	*nhr-49*	102/113	60.24	1.52	0.0001	
AGP53	*nhr-49 glmEx11 [Pcol-12::nhr-49::GFP + Pmyo-2::mCh]*	28/32	48.62	4.65	0.0001	0.146

Table S4e.2: SURVIVAL ON OP50

Strain	Background/Genotype	Trial 1@ n = obs/ total	Mean (h)	SE +/-	Bonferroni P-value P (*vs* N2)	P (*vs nhr 49*)
WT	Wildtype	67/73	16.67	0.65		
AGP12a	*nhr-49*	33/77	14.39	0.69	0.1514	
AGP53	*nhr-49 glmEx11 [Pcol-12::nhr-49::GFP + Pmyo-2::mCh]*	59/70	16.89	0.65	1	0.0926
		**Trial 2&**				
WT	Wildtype	89 / 122	15.42	0.49		
AGP12a	*nhr-49*	110 / 119	12.5	0.36	<0.0001	
AGP53	*nhr-49 glmEx11 [Pcol-12::nhr-49::GFP + Pmyo-2::mCh]*	131 / 141	14.86	0.45	1	<0.0001

*, #, ^, $, & on different tabs/sub-sections of Table S4 denote trials performed at the same time

Underlined Trials shown in Fig.4

Table S5a: Impact of endogenous promoter-driven NHR-49 overexpression on survival of wild-type *C. elegans* on PA14 (a.1) and OP50 (a.2)

Table S5a. 1: SURVIVAL ON PA14

Strain	Background/Genotype	Trial 1*			Bonferroni P-value	Non-Bonferroni P-value
		n = obs/ total	Mean (h)	SE +/-		
WT	Wild type	126 / 145	54.32	1.09		
AGP24f	*glmEx5 [Pnhr-49::nhr-49::GFP + Pmyo-2::mCh]*	123 / 153	68.14	1.7	<0.0001	<0.0001
**Trial 2#**						
WT	Wild type	60 / 117	78.4	2.66		
AGP24f	*glmEx5 [Pnhr-49::nhr-49::GFP + Pmyo-2::mCh]*	61 / 110	71.05	3.78	0.229	0.0286
**Trial 3^**						
WT	Wild type	102 / 129	76.98	2.25		
AGP24f	*glmEx5 [Pnhr-49::nhr-49::GFP + Pmyo-2::mCh]*	75 / 121	76.89	3.06	1	0.895

Table S5a.2: SURVIVAL ON OP50

Strain	Background/Genotype	Trial 1@			Bonferroni P-value (*vs* N2)	Non-Bonferroni P (*vs* N2)
		n = obs/ total	Mean (h)	SE +/-		
WT	Wild type	67 / 73	16.67	0.65		
AGP12a	*nhr-49*	33 / 77	14.39	0.69	0.1514	
AGP24f	*glmEx5 [Pnhr-49::nhr-49::GFP + Pmyo-2::mCh]*	35 / 44	20.83	1.01	0.0109	0.0016
**Trial 2~**						
WT	Wild type	73 / 121	17.81	0.56		
AGP12a	*nhr-49*	69 / 76	14.6	0.54	0.0003	
AGP24f	*glmEx5 [Pnhr-49::nhr-49::GFP + Pmyo-2::mCh]*	70 / 115	17.58	0.75	1	0.603

*, #, ^, &, @, ~ on different tabs/sub-sections of Table S5 denote trials performed at the same time
Underlined Trials shown in Fig. 5

Table S5b: Impact of neuronal (*unc-119*) promoter-driven NHR-49 overexpression on survival of wild-type *C. elegans* on PA14 (b.1) and OP50 (b.2)

Table S5B. 1: SURVIVAL ON PA14						
Strain	Background/Genotype	Trial 1*			Bonferroni P-value	Non-Bonferroni P-value
		n = obs/ total	Mean (h)	SE +/-		
WT	Wild type	126 / 145	54.32	1.09		
AGP102	*glmEx20 [Pnhr-49::unc-119::GFP + Pmyo-2::mCh]*	114 / 136	73.72	1.61	<0.0001	<0.0001
Trial 2#						
WT	Wild type	60 / 117	78.4	2.66		
AGP102	*glmEx20 [Pnhr-49::unc-119::GFP + Pmyo-2::mCh]*	88/120	70.79	2.11	0.093	0.0116
Trial 3^						
WT	Wild type	102 / 129	76.98	2.25		
AGP102	*glmEx20 [Pnhr-49::unc-119::GFP + Pmyo-2::mCh]*	96/127	86.37	2.44	0.0333	0.0135

Table S5B.2: SURVIVAL ON OP50						
Strain	Background/Genotype	Trial 1@			Bonferroni P-value	Non-Bonferroni P-value
		n = obs/ total	Mean (h)	SE +/-		
WT	Wild type	67 / 73	16.67	0.65		
AGP102	*glmEx20 [Pnhr-49::unc-119::GFP + Pmyo-2::mCh]*	39/57	19.31	0.75	0.0651	0.0093
Trial 2						
WT	Wild type	74 / 91	24.2	0.55		
AGP102	*glmEx20 [Pnhr-49::unc-119::GFP + Pmyo-2::mCh]*	57 / 94	22.35	0.6	0.4163	0.0416
Trial 3						
WT	Wild type	71 / 100	23.8	0.58		
AGP102	*glmEx20 [Pnhr-49::unc-119::GFP + Pmyo-2::mCh]*	40 / 71	21.53	0.81	0.074	0.0092
Trial 4						
WT	Wildtype	71 / 82	17.99	0.58		
AGP102	*glmEx20 [Pnhr-49::unc-119::GFP + Pmyo-2::mCh]*	32 / 70	23.23	0.97	<0.0001	<0.0001

*, #, ^, &, @, ~ on different tabs/sub-sections of Table S5 denote trials performed at the same time
Underlined Trials shown in Fig. 5

Table S5c: Impact of Intestinal (*gly-19*) promoter-driven NHR-49 overexpression on survival of wild-type *C. elegans* on PA14 (c.1) or OP50 (c.2)

Table S5c. 1: SURVIVAL ON PA14

Strain	Background/Genotype	Trial 1*			Bonferroni P-value	Non-Bonferroni P-value
		n = obs/ total	Mean (h)	SE +/-		
WT	Wild type	126 / 145	54.32	1.09		
AGP40	*glmEx9 [Pgly-19::nhr-49::GFP + Pmyo-2::mCherry]*	117 / 131	73.6	1.83	<0.0001	<0.0001
**Trial 2#**						
WT	Wild type	60/117	78.4	2.66		
AGP40	*glmEx9 [Pgly-19::nhr-49::GFP + Pmyo-2::mCherry]*	92/128	94.83	2.72	0.0007	0.0001
**<u>Trial 3^</u>**						
WT	Wildtype	102/129	76.98	2.25		
AGP40	*glmEx9 [Pgly-19::nhr-49::GFP + Pmyo-2::mCherry]*	96/124	91.97	2.65	0.0002	0.0001

Table S5c.2: SURVIVAL ON OP50

Strain	Background Genotype	Trial 1@			Bonferroni P-value (*vs* N2)	Non-Bonferroni P (*vs* N2)
		n = obs/ total	Mean (days)	SE +/-		
WT	Wild type	67/73	16.67	0.65		
AGP12a	*nhr-49*	33/77	14.39	0.69	0.1514	
AGP40	*glmEx9 [Pgly-19::nhr-49::GFP + Pmyo-2::mCherry]*	64/67	18.49	0.82	0.6491	0.0927
**<u>Trial 2~</u>**						
WT	Wild type	73 / 121	17.81	0.56		
AGP12a	*nhr-49*	69 / 76	14.6	0.54	0.0003	
AGP40	*glmEx9 [Pgly-19::nhr-49::GFP + Pmyo-2::mCherry]*	86 / 116	19.06	0.4	1	0.202

*, #, ^, &, @, ~ on different tabs/sub-sections of Table S5 denote trials performed at the same time
<u>Underlined Trials</u> shown in Fig. 5

Table S5d: Impact of muscle (*myo-3*) promoter-driven NHR-49 overexpression on survival of wild-type *C. elegans* on PA14 (d.1) or OP50 (d.2)

Table S5d. 1: SURVIVAL ON PA14

Strain	Background/Genotype	Trial 1*			Bonferroni P-value	Non-Bonferroni P-value
		n = obs/ total	Mean (h)	SE +/-		
WT	Wild type	126 / 145	54.32	1.09		
AGP27	*glmEx9 [Pmyo-3::nhr-49 ::GFP + Pmyo-2::mCherry]*	106 / 126	61.12	2.11	0.0007	0.0001
**Trial 2#**						
WT	Wild type	60/117	78.4	2.66		
AGP27	*glmEx9 [Pmyo-3::nhr-49 ::GFP + Pmyo-2::mCherry]*	61/100	72.85	4.25	1	0.289
**Trial 3^**						
WT	Wild type	102/129	76.98	2.25		
AGP27	*glmEx9 [Pmyo-3::nhr-49 ::GFP + Pmyo-2::mCherry]*	66/100	72.11	4.28	1	0.363

Table S5d.2: SURVIVAL ON OP50

Strain	Background Genotype	Trial 1@			Bonferroni P-value (*vs* N2)	Non-Bonferroni P (*vs* N2)
		n = obs/ total	Mean (days)	SE +/-		
WT	Wild type	67/73	16.67	0.65		
AGP12a	*nhr-49*	33/77	14.39	0.69	0.1514	
AGP27	*glmEx9 [Pmyo-3::nhr-49 ::GFP + Pmyo-2::mCherry]*	39/52	14.6	0.55	0.5331	0.0762
**Trial 2~**						
WT	Wild type	73 / 121	17.81	0.56		
AGP12a	*nhr-49*	69 / 76	14.6	0.54	0.0003	
AGP27	*glmEx9 [Pmyo-3::nhr-49 ::GFP + Pmyo-2::mCherry]*	84 / 109	19.89	0.68	0.0389	0.0065

*, #, ^, &, @, ~ on different tabs/sub-sections of Table S5 denote trials performed at the same time
Underlined Trials shown in Fig. 5

Table S5e: Impact of hypodermal *(col-12)* promoter-driven NHR-49 overexpression on survival of wild-type *C. elegans* on PA14 (e.1) or OP50 (e.2)

Table S5e. 1: SURVIVAL ON PA14

Strain	Background/Genotype	Trial 1*			Bonferroni P-value	Non-Bonferroni P-value
		n = obs/ total	Mean (h)	SE +/-		
WT	Wild type	126 / 145	54.32	1.09		
AGP45	*glmEx11 [Pcol-12::nhr-49::GFP + Pmyo-2::mCherry]*	117 / 137	77.16	1.44	<0.0001	<0.0001
**Trial 2#**						
WT	Wild type	60/117	78.4	2.66		
AGP45	*glmEx11 [Pcol-12::nhr-49::GFP + Pmyo-2::mCherry]*	77/125	85.91	3.67	1	0.326
**<u>Trial 3^</u>**						
WT	Wildtype	102/129	76.98	2.25		
AGP45	*glmEx11 [Pcol-12::nhr-49::GFP + Pmyo-2::mCherry]*	63/97	82.68	2.65	0.3504	0.013

Table S5e.2: SURVIVAL ON OP50

Strain	Background Genotype	Trial 1@			Bonferroni P-value (*vs* N2)	Non-Bonferroni P (*vs* N2)
		n = obs/ total	Mean (days)	SE +/-		
WT	Wild type	67/73	16.67	0.65		
AGP12a	*nhr-49*	33/77	14.39	0.69	0.1514	
AGP45	*glmEx11 [Pcol-12::nhr-49::GFP + Pmyo-2::mCherry]*	57/66	17.27	0.67	1	0.4482
**<u>Trial 2~</u>**						
WT	Wild type	73 / 121	17.81	0.56		
AGP12a	*nhr-49*	69 / 76	14.6	0.54	0.0003	
AGP45	*glmEx11 [Pcol-12::nhr-49::GFP + Pmyo-2::mCherry]*	95 / 120	18.3	0.53	1	0.468

*, #, ^, &, @, ~ on different tabs/sub-sections of Table S5 denote trials performed at the same time

<u>Underlined Trials</u> shown in Fig. 5

Table S6a: Impact of tissue-specific expression of *nhr-49(et7)* on survival on PA14 (a.1) and lifespan on OP50 (a.2)

Table S6a.1: SURVIVAL ON PA14

Tissue of Expression	Background / Genotype	Trial 1#			Bonferroni P-value	
		n = obs/ total	Mean (h)	SE +/-	P (vs N2)	P (vs *nhr-49*)
	Wildtype	87 / 150	77.36	1.79		
	nhr-49(nr2041) lof	116 / 150	62.34	1.07	<0.0001	
Neurons	*Punc-119::nhr-49(et7)::GFP + Pmyo-2::mCherry*	68 / 125	67.18	1.46	0.0003	0.026
Hypodermis	*Pcol-12::nhr-49(et7)::GFP + Pmyo-2::mCherry*	73 / 125	74.5	2.4	1	<0.0001
Neurons	*nhr-49;[Punc-119::nhr-49(et7)::GFP + Pmyo-2::mCherry]*	72 / 115	63.45	1.57	<0.0001	1
**Trial 2~**						
	Wildtype	94 / 120	75.16	2.74		
	nhr-49(nr2041) lof	117 / 130	53.73	1.54	<0.0001	
Neurons	*Punc-119::nhr-49(et7)::GFP + Pmyo-2::mCherry*	98 / 125	62.92	1.59	0.0017	0.0037
Hypodermis	*Pcol-12::nhr-49(et7)::GFP + Pmyo-2::mCherry*	118 / 145	55.46	2.11	<0.0001	1
Neurons	*nhr-49;[Punc-119::nhr-49(et7)::GFP + Pmyo-2::mCherry]*	123 / 136	58.22	1.12	<0.0001	0.59
**Trial 3$**						
	Wildtype	78 / 106	67.46	2.15		
	nhr-49(nr2041) lof	94 / 105	52.23	1.69	> 0.0001	
Neurons	*Punc-119::nhr-49(et7)::GFP + Pmyo-2::mCherry*	73 / 107	57.15	1.71	0.0002	0.0197
Hypodermis	*Pcol-12::nhr-49(et7)::GFP + Pmyo-2::mCherry*	69 / 105	60.24	2.22	0.0647	0.0013
Neurons	*nhr-49;[Punc-119::nhr-49 (et7)::GFP + Pmyo-2::mCherry]*	89 / 111	61.77	1.71	0.0316	> 0.0001

Table S6a.2: SURVIVAL ON OP50

Tissue of Expression	Background / Genotype	Trial 1◇			Bonferroni P-value	
		n = obs/ total	Mean (h)	SE +/-	P (vs N2)	P (vs *nhr-49*)
	Wildtype	99 / 120	15.61	0.46		
	nhr-49(nr2041) lof	101 / 119	7.66	0.2	< 0.0001	
Neurons	*Punc-119::nhr-49(et7)::GFP + Pmyo-2::mCherry*	61 / 80	12.63	0.76	0.023	< 0.0001
Hypodermis	*Pcol-12::nhr-49(et7)::GFP + Pmyo-2::mCherry*	82 / 100	11.11	0.63	< 0.0001	< 0.0001
Neurons	*nhr-49;[Punc-119::nhr-49(et7)::GFP + Pmyo-2::mCherry]*	69 / 89	9.31	0.38	< 0.0001	0.0004
**Trial 2†**						
	Wildtype	103 / 136	16.49	0.44		
	nhr-49(nr2041) lof	119 / 130	7.31	0.18	< 0.0001	
Neurons	*Punc-119::nhr-49(et7)::GFP + Pmyo-2::mCherry*	28 / 60	10.23	0.74	< 0.0001	< 0.0001
Hypodermis	*Pcol-12::nhr-49(et7)::GFP + Pmyo-2::mCherry*	60 / 90	9.09	0.53	< 0.0001	0.0003
Neurons	*nhr-49;[Punc-119::nhr-49(et7)::GFP + Pmyo-2::mCherry]*	98 / 134	8.75	0.23	< 0.0001	< 0.0001

Special characters #, ~, $, ◇, † on trial headings denote the trial was performed at the same time as correspondingly-labeled trials in Table S2A. Underlined Trials shown in Fig. 6.

Table S6b: Impact of Fenofibrate supplementation on PA14 survival of *nhr-49* mutants expressing NHR-49 in muscles (*myo-3*) or hypodermis (*col-12*)

SURVIVAL ON PA14						
**Tissue of Expression**	**Genotype**	**Treatment**	**Trial 1**			**Bonferroni P[illegible] value**
			**n = obs/ total**	**Mean (h)**	**SE +/-**	**P (vs Control)**
Muscle	*nhr-49;[Pmyo-3::nhr-49::GFP + Pmyo-2::mCherry]*	Control	62 / 88	57.88	1.55	
		Fenofibrate	66 / 100	59.35	1.36	1
Hypodermis	*nhr-49;[Pcol-12::nhr-49::GFP + Pmyo-2::mCherry]*	Control	100 / 133	63.59	0.93	
		Fenofibrate	111 / 135	62.56	1.07	1
**Trial 2**						
Muscle	*nhr-49;[Pmyo-3::nhr-49::GFP + Pmyo-2::mCherry]*	Control	57 / 71	52.76	1.48	
		Fenofibrate	117 / 120	55.35	1.29	0.6649
Hypodermis	*nhr-49;[Pcol-12::nhr-49::GFP + Pmyo-2::mCherry]*	Control	94 / 120	61.2	1.37	
		Fenofibrate	89 / 105	64.22	1.24	1
**Trial 3**						
Muscle	*nhr-49;[Pmyo-3::nhr-49::GFP + Pmyo-2::mCherry]*	Control	43 / 45	60.47	2.38	
		Fenofibrate	54 / 58	66.54	1.53	0.6314
Hypodermis	*nhr-49;[Pcol-12::nhr-49::GFP + Pmyo-2::mCherry]*	Control	63 / 81	68.34	1.46	
		Fenofibrate	126 / 154	68.89	1.07	1
**Trial 4**						
Muscle	*nhr-49;[Pmyo-3::nhr-49::GFP + Pmyo-2::mCherry]*	Control	132 / 150	60.41	1.07	
		Fenofibrate	127 / 149	59.22	1	1
Hypodermis	*nhr-49;[Pcol-12::nhr-49::GFP + Pmyo-2::mCherry]*	Control	114 / 152	61.42	1.42	
		Fenofibrate	130 / 151	58.43	1.03	1
**Trial 5**						
Muscle	*nhr-49;[Pmyo-3::nhr-49::GFP + Pmyo-2::mCherry]*	Control	135/150	55.43	1.08	
		Fenofibrate	124/153	52.82	1.05	0.9918
Hypodermis	*nhr-49;[Pcol-12::nhr-49::GFP + Pmyo-2::mCherry]*	Control	112/151	55.04	1.24	
		Fenofibrate	120/150	53.38	1.12	1

Table S7a: Impact of endogenous promoter-driven NHR-49 expression on survival of *glp-1* mutants on PA14 (a.1) and OP50 (a.2)

Table S7a. 1: SURVIVAL ON PA14						
Strain	Background/Genotype	Trial 1*			Bonferroni P-value	
		n = obs/ total	Mean (h)	SE +/-	P (*vs* N2)	P (*vs* *glp-1*)
WT	Wildtype	83 / 105	67.31	1.37		
CF1903	*glp-1*	116 / 127	83.68	2.04	<0.0001	
AGP29	*glp-1 glmEx6 [Pnhr-49::nhr-49 ::GFP + Pmyo-2::mCh]*	105 / 112	38.6	1.57	<0.0001	<0.0001
**Trial 2#**						
WT	Wildtype	61/112	72.21	3.63		
CF1903	*glp-1*	95/109	88.89	2.57	0.001	
AGP29	*glp-1 glmEx6 [Pnhr-49::nhr-49 ::GFP + Pmyo-2::mCh]*	98/115	44.15	1.92	<0.0001	<0.0001
**<u>Trial 3^</u>**						
WT	Wildtype	68 / 120	68.39	2.2		
CF1903	*glp-1*	102 / 117	94.91	3.2	0.0001	
AGP29	*glp-1 glmEx6 [Pnhr-49::nhr-49 ::GFP + Pmyo-2::mCh]*	94 / 105	39.35	1.56	0.0001	0.0001
**Trial 4**						
WT	Wildtype	88 / 128	92.05	1.99		
CF1903	*glp-1*	101 / 131	106.42	2.74	0.0004	
AGP29	*glp-1 glmEx6 [Pnhr-49::nhr-49 ::GFP + Pmyo-2::mCh]*	132 / 156	53.58	2.18	<0.0001	<0.0001

Table S7a.2: SURVIVAL ON OP50						
Strain	Background/Genotype	Trial 1@			Bonferroni P-value	
		n = obs/ total	Mean (h)	SE +/-	P (*vs* N2)	P (*vs* *glp-1*)
AGP22	*nhr-49;glp-1*	72/72	11.27	0.26		<0.0001
CF1903	*glp-1*	65/77	25.78	0.98		
AGP29	*glp-1 glmEx6 [Pnhr-49::nhr-49 ::GFP + Pmyo-2::mCh]*	13/14	22.62	1.85		0.0755
**<u>Trial 2~</u>**						
WT	Wildtype	82 / 122	15.53	0.58		
CF1903	*glp-1*	112 / 124	22.98	0.67	<0.0001	
AGP29	*glp-1 glmEx6 [Pnhr-49::nhr-49 ::GFP + Pmyo-2::mCh]*	94 / 108	21.31	0.42	<0.0001	0.003
**Trial 3$**						
WT	Wildtype	73 / 121	17.81	0.56		
CF1903	*glp-1*	101 / 114	23.35	0.69	<0.0001	
AGP29	*glp-1 glmEx6 [Pnhr-49::nhr-49 ::GFP + Pmyo-2::mCh]*	55 / 120	21.71	0.53	<0.0001	0.3082

Special characters *, #, ^, &, @, ~ on different tabs/sub-sections of Table S7 denote trials performed at the same time. <u>Underlined</u> Trials shown in Fig. S5

Table S7b: Impact of neuronal (*unc-119*) promoter-driven NHR-49 expression on survival of *glp-1* mutants on PA14 (b.1) and OP50 (b.2)

Strain	Background/Genotype	Trial 1*			Bonferroni P-value	
		n = obs/ total	Mean (h)	SE +/-	P (*vs* N2)	P (*vs glp 1*)
**Table S7a.1: SURVIVAL ON PA14**						
WT	Wildtype	83 / 105	67.31	1.37		
CF1903	*glp-1*	116 / 127	83.68	2.04	<0.0001	
AGP105	*glp-1 glmEx20 [Punc-119::nhr-49::GFP + Pmyo-2::mCh]*	97 / 117	70.81	2.96	0.0174	0.242
**Trial 2#**						
WT	Wildtype	61/112	72.21	3.63		
CF1903	*glp-1*	95/109	88.89	2.57	0.001	
AGP105	*glp-1 glmEx20 [Punc-119::nhr-49::GFP + Pmyo-2::mCh]*	111/120	59.86	3.3	0.1603	<0.0001
**Trial 3^**						
WT	Wildtype	68 / 120	68.39	2.2		
CF1903	*glp-1*	102 / 117	94.91	3.2	0.0001	
AGP105	*glp-1 glmEx20 [Punc-119::nhr-49::GFP + Pmyo-2::mCh]*	73 / 80	73.22	4.93	1	0.0582

Table S7b.2: SURVIVAL ON OP50

Strain	Background/Genotype	Trial 1			Bonferroni P-value	
		n = obs/ total	Mean (h)	SE +/-	P (*vs* N2)	P (*vs glp 1*)
WT	Wildtype	52/71	16.26	0.91		
CF1903	*glp-1*	51/70	24.43	1.08	<0.0001	
AGP105	*glp-1 glmEx20 [Punc-119::nhr-49::GFP + Pmyo-2::mCh]*	29 / 42	21.78	1.86	0.01	1
**Trial 2**						
WT	Wildtype	74 / 91	24.2	0.55		
CF1903	*glp-1*	120 / 124	30.54	0.79	<0.0001	
AGP105	*glp-1 glmEx20 [Punc-119::nhr-49::GFP + Pmyo-2::mCh]*	70 / 80	31.4	1.1	<0.0001	1
**Trial 3**						
WT	Wildtype	71 / 100	23.8	0.58		
CF1903	*glp-1*	83 / 84	22.58	1.06	1	
AGP105	*glp-1 glmEx20 [Punc-119::nhr-49::GFP + Pmyo-2::mCh]*	51 / 63	26.69	0.74	0.1157	1
**Trial 4**						
WT	Wildtype	71 / 82	17.99	0.58		
CF1903	*glp-1*	109 / 121	24.25	0.91	<0.0001	
AGP105	*glp-1 glmEx20 [Punc-119::nhr-49::GFP + Pmyo-2::mCh]*	99 / 120	28.14	0.89	<0.0001	0.38

Special characters *, #, ^, &, @, ~ on different tabs/sub-sections of Table S7 denote trials performed at the same time. Underlined Trials shown in Fig. S5

Table S7c: Impact of intestinal (*gly-19*) promoter-driven NHR-49 expression on survival of *glp-1* mutants on PA14 (c.1) and OP50 (c.2)

Table S7c. 1: SURVIVAL ON PA14

Strain	Background/Genotype	Trial 1*			Bonferroni P-value	
		n = obs/ total	Mean (h)	SE +/-	P (*vs* N2)	P (*vs glp-1*)
WT	Wildtype	83 / 105	67.31	1.37		
CF1903	*glp-1*	116 / 127	83.68	2.04	<0.0001	
AGP44	*glp-1 glmEx10 [Pgly-19::nhr-49::GFP + Pmyo-2::mCh]*	89 / 108	80.91	2.67	<0.0001	1
**Trial 2#**						
WT	Wildtype	61/112	72.21	3.63		
CF1903	*glp-1*	95/109	88.89	2.57	0.001	
AGP44	*glp-1 glmEx10 [Pgly-19::nhr-49::GFP + Pmyo-2::mCh]*	106/120	69.98	2.52	1	<0.0001
**Trial 3^**						
WT	Wildtype	68 / 120	68.39	2.2		
CF1903	*glp-1*	102 / 117	94.91	3.2	0.0001	
AGP44	*glp-1 glmEx10 [Pgly-19::nhr-49::GFP + Pmyo-2::mCh]*	97 / 115	82.13	2.71	0.0044	0.0086

Table S7c.2: SURVIVAL ON OP50

Strain	Background/Genotype	Trial 1@			Bonferroni P-value	
		n = obs/ total	Mean (h)	SE +/-	P (*vs* N2)	P (*vs glp-1*)
AGP22	*nhr-49;glp-1*	72/72	11.27	0.26		<0.0001
CF1903	*glp-1*	65/77	25.78	0.98		
AGP44	*glp-1 glmEx10 [Pgly-19::nhr-49::GFP + Pmyo-2::mCh]*	26/27	20.06	1.09		0.0003
**Trial 2~**						
WT	Wildtype	82 / 122	15.53	0.58		
CF1903	*glp-1*	112 / 124	22.98	0.67	<0.0001	
AGP44	*glp-1 glmEx10 [Pgly-19::nhr-49::GFP + Pmyo-2::mCh]*	105 / 122	20.69	0.44	<0.0001	0.0002
**Trial 3$**						
WT	Wildtype	73 / 121	17.81	0.56		
CF1903	*glp-1*	101 / 114	23.35	0.69	<0.0001	
AGP44	*glp-1 glmEx10 [Pgly-19::nhr-49::GFP + Pmyo-2::mCh]*	101 / 116	20.93	0.41	0.0004	0.0004

Special characters *, #, ^, &, @, ~ on different tabs/sub-sections of Table S7 denote trials performed at the same time. Underlined Trials shown in Fig. S5

Table S7d: Impact of muscle (*myo-3*) promoter-driven NHR-49 expression on survival of *glp-1* mutants on PA14 (d.1) and OP50 (d.2)

Table S7d. 1: SURVIVAL ON PA14

Strain	Background/Genotype	Trial 1*			Bonferroni P-value	
		n = obs/ total	Mean (h)	SE +/-	P (*vs* N2)	P (*vs glp-1*)
WT	Wildtype	83 / 105	67.31	1.37		
CF1903	*glp-1*	116 / 127	83.68	2.04	<0.0001	
AGP36	*glp-1 glmEx7 [Pmyo-3::nhr-49::GFP + Pmyo-2::mCh]*	96 / 118	73.05	2.12	0.0378	0.0107
**Trial 2#**						
WT	Wildtype	61/112	72.21	3.63		
CF1903	*glp-1*	95/109	88.89	2.57	0.001	
AGP36	*glp-1 glmEx7 [Pmyo-3::nhr-49::GFP + Pmyo-2::mCh]*	110/120	80.21	2.84	0.3629	0.6544
**Trial 3^**						
WT	Wildtype	68 / 120	68.39	2.2		
CF1903	*glp-1*	102 / 117	94.91	3.2	0.0001	
AGP36	*glp-1 glmEx7 [Pmyo-3::nhr-49::GFP + Pmyo-2::mCh]*	110 / 120	88.62	2.93	<0.0001	0.747

Table S7d.2: SURVIVAL ON OP50

Strain	Background/Genotype	Trial 1@			Bonferroni P-value	
		n = obs/ total	Mean (h)	SE +/-	P (*vs* N2)	P (*vs glp-1*)
AGP22	*nhr-49;glp-1*	72/72	11.27	0.26		<0.0001
CF1903	*glp-1*	65/77	25.78	0.98		
AGP36	*glp-1 glmEx7 [Pmyo-3::nhr-49::GFP + Pmyo-2::mCh]*	46/83	24.65	0.98		1
**Trial 2$**						
WT	Wildtype	73 / 121	17.81	0.56		
CF1903	*glp-1*	101 / 114	23.35	0.69	<0.0001	
AGP36	*glp-1 glmEx7 [Pmyo-3::nhr-49::GFP + Pmyo-2::mCh]*	104 / 121	21.89	0.55	<0.0001	0.3937

Special characters *, #, ^, &, @, ~ on different tabs/sub-sections of Table S7 denote trials performed at the same time. Underlined Trials shown in Fig. S5

Table S7e: Impact of hypodermal (*col-12*) promoter-driven NHR-49 expression on survival of *glp-1* mutants on PA14 (e.1) and OP50 (e.2)

Table S7e.1: SURVIVAL ON PA14						
**Strain**	**Background/Genotype**	**Trial 1***			**Bonferroni P-value**	
		**n = obs/ total**	**Mean (h)**	**SE +/-**	**P (*vs* N2)**	**P (*vs glp-1*)**
WT	Wildtype	83 / 105	67.31	1.37		
CF1903	*glp-1*	116 / 127	83.68	2.04	<0.0001	
AGP41	*glp-1 glmEx11 [Pcol-12::nhr-49::GFP + Pmyo-2::mCh]*	80 / 98	69.92	2.58	0.0674	0.009
**Trial 2#**						
WT	Wildtype	61/112	72.21	3.63		
CF1903	*glp-1*	95/109	88.89	2.57	0.001	
AGP36	*glp-1 glmEx7 [Pmyo-3::nhr-49::GFP + Pmyo-2::mCh]*	12./13	79.72	9.95	1	1
**<u>Trial 3^</u>**						
WT	Wildtype	68 / 120	68.39	2.2		
CF1903	*glp-1*	102 / 117	94.91	3.2	0.0001	
AGP41	*glp-1 glmEx11 [Pcol-12::nhr-49::GFP + Pmyo-2::mCh]*	47 / 60	65.17	4.78	1	<0.0001

Table S7e.2: SURVIVAL ON OP50						
**Strain**	**Background/Genotype**	**Trial 1@**			**Bonferroni P-value**	
		**n = obs/ total**	**Mean (h)**	**SE +/-**	**P (*vs* N2)**	**P (*vs glp-1*)**
AGP22	*nhr-49;glp-1*	72/72	11.27	0.26		<0.0001
CF1903	*glp-1*	65/77	25.78	0.98		
AGP41	*glp-1 glmEx11 [Pcol-12::nhr-49::GFP + Pmyo-2::mCh]*	28/42	24.06	0.83		0.0611
**<u>Trial 2$</u>**						
WT	Wildtype	73 / 121	17.81	0.56		
CF1903	*glp-1*	101 / 114	23.35	0.69	<0.0001	
AGP41	*glp-1 glmEx11 [Pcol-12::nhr-49::GFP + Pmyo-2::mCh]*	84 / 102	23.79	0.54	<0.0001	1

Special characters *, #, ^, &, @, ~ on different tabs/sub-sections of Table S7 denote trials performed at the same time. <u>Underlined</u> Trials shown in Fig. S5

Table S8: Comparison of lipid-catabolic genes regulated by NHR-49 in *glp-1* mutants and upon PA14 infection							
**Lipolysis Process**	**Gene**	**Wormbase Gene ID**	**Impact of Germline Loss [dependence on DAF-16 (D16), TCER-1 (T1) *(Amrit et al., 2016)* &/or NHR-49 (N49 *(Ratnappan et al., 2014)*]**	**Studies documenting PA14-induced gene-expression changes**			
				Troemel et al., 2006	***Dasgupta et al., 2020* (only UP reported)**	***Twumasi-Boateng & Shapira, 2012***	***Fletcher et al., 2019***
**Mitochondrial β-oxidation**	*acs-2*	WBGene00009221	UP (D16, T1, N49)	UP	UP		
	acs-22	WBGene00017012	UP (T1, N49)				DOWN
	cpt-5	WBGene00008629	UP (N49)				DOWN
	acdh-1	WBGene00016943	UP (N49)	DOWN	DOWN		DOWN
	acdh-2	WBGene00015894	UP (N49)				DOWN
	acdh-9	WBGene00017874	DOWN (T1, N49)				
	acdh-11	WBGene00012860	UP (T1, N49)				DOWN
	ech-1.1	WBGene00001150	UP (N49)				
	ech-7	WBGene00001156	UP (D16, T1, N49)				DOWN
	hacd-1	WBGene00019978	UP (D16, T1, N49)	DOWN		DOWN	DOWN
	B0272.3	WBGene00007129	UP (N49)				
	acaa-2	WBGene00009952	UP (D16, T1, N49)				DOWN
	kat-1	WBGene00002183	UP (N49)				DOWN
**Peroxisomal β-Oxidation**	*F08A8.2*	WBGene00008565	UP (D16, T1, N49)	DOWN			
	F08A8.3	WBGene00008566	UP (T1, N49)	DOWN		DOWN	
	F08A8.4/a	WBGene00008567	UP (T1, N49)	DOWN		DOWN	
	acox-1.1	WBGene00008564	UP (D16, T1, N49)	UP	UP	UP	UP
	ech-9	WBGene00001158	DOWN (N49)	UP	UP	UP	UP
**Lipases and Lipase-like Proteins**	*lipl-1*	WBGene00010062	UP (D16, T1, N49)	UP	UP		UP
	lipl-2	WBGene00009773	UP (D16, T1, N49)		UP		
	lipl-3	WBGene00020016	UP (N49)				DOWN
	lipl-4	WBGene00019376	UP (N49)				
	lipl-5	WBGene00022642	UP (D16, T1, N49)	DOWN		DOWN	DOWN
	lipl-6	WBGene00021963	UP (N49)				
	lips-5	WBGene00011725	UP (N49)				UP
	lips-14	WBGene00019208	UP (D16, T1, N49)	DOWN			DOWN
	lips-17	WBGene00019939	UP (N49)				
	fil-1	WBGene00011321	DOWN (N49)	UP	UP		UP
UP: Gene upregulated; DOWN: Gene downregulated; Genes similarly changed in *glp-1* vs. PA14 are highlighted in green and oppositely changes ones in red.							

Table S9: Impact of RNAi knockdown of NHR-49-target lipid metabolic genes and partner NHRs on survival of wildtype animals on PA14

RNAi treatment	Trial 1			P-value
	n = obs/ total	Mean (h)	SE +/-	P (*vs* Control)
Control	100 / 130	63.8	1.51	
nhr-80	82 / 131	57.9	1.35	0.003
nhr-71	111 / 143	70.1	1.55	0.005
nhr-13	104 / 124	61.14	1.19	0.093
acox-1.1	99 / 114	66.81	1.13	0.445
hacd-1	89 / 133	61.39	1.31	0.147
**Trial 2**				
Control	96 / 125	65.56	1.26	
nhr-80	103 / 125	62.2	1.26	0.062
nhr-71	88 / 125	67.6	1.57	0.188
nhr-13	95 / 125	65.89	1.16	0.909
acox-1.1	95 / 105	66.45	1.57	0.475
hacd-1	88 / 107	70.05	1.88	0.016

Table S10. Strains used in this study.

Strain	Genotype	Source
N2	Wild type, Ghazi Lab	
CF1903	*glp-1(e2144ts)*	
AGP12a	*nhr-49(nr2041)I* Outcrossed Ghazi Lab N2 3x	
AGP22	*nhr-49(nr2041)I; glp-1(e2141ts)III*	
AGP110	*nhr-49(et7).* Outcrossed *nhr-49(et7)* to Ghazi Lab N2 4x	This study
VC1668	*fmo-2(ok2147)*	CGC
RB1899	*acs-2 (ok2457)*	CGC
EB271	*fmo-2p::gfp + rol-6(su1006)*	CGC
WBM170	*wbmEx57 [acs-2p::GFP + rol-6(su1006)]*	*Burkewitz et al., 2015*
AGP309	*glp-1(e2144ts);fmo-2(ok2147)*	This study
AGP308	*glp-1(e2144ts);acs-2(ok2457)*	This study
**NHR-49 Transgenic Strains**		
**Wild Type Background**		
AGP24f	*glmEx5[Pnhr-49::nhr-49::gfp + Pmyo-2::mCherry]*	*Ratnappan et al., 2014*
AGP27a	*glmEx7[Pmyo-3::nhr-49::GFP + Pmyo-2::mCherry]*	This study
AGP40a	*glmEx9[Pgly-19::nhr-49::GFP + Pmyo-2::mCherry]*	This study
AGP45	*glmEx11[Pcol-12::nhr-49::GFP + Pmyo-2::mCherry]*	This study
AGP102a	*glmEx20[Punc-119::nhr-49::GFP + Pmyo-2::mCherry]*	This study
nhr-49 **Mutant Background**		
AGP33a	*nhr-49(nr2041)I;glmEx5 [Pnhr-49::nhr-49::GFP + Pmyo-2::mCherry]*	This study
AGP63	*nhr-49(nr2041)I;glmEx8 [Pmyo-3::nhr-49::GFP + Pmyo-2::mCherry]*	This study
AGP65	*nhr-49(nr2041)I;glmEx9 [Pgly-19::nhr-49::GFP + Pmyo-2::mCherry]*	This study
AGP53	*nhr-49(nr2041)I;glmEx11 [Pcol-12::nhr-49::GFP + Pmyo-2::mCherry]*	This study
AGP103a	*nhr-49(nr2014)I;glmEx20[Punc-119::nhr-49::GFP + Pmyo-2::mCherry]*	This study
glp-1 **Mutant Background**		
AGP29d	*glp-1(e2141ts)III;glmEx6[Pnhr-49::nhr-49::GFP + Pmyo-2::mCherry].* Obtained by crossing AGP28a x CF1903.	This study
AGP36a	*glp-1(e2141ts)III;glmEx7[Pmyo-3::nhr-49::GFP + Pmyo-2::mCherry]*	This study
AGP44	*glp-1(e2141ts)III;glmEx10 [Pgly-19::nhr-49::GFP + Pmyo-2::mCherry]*	This study
AGP41a/b	*glp-1(e2141ts) III;glmEx11[Pcol-12::nhr-49::GFP + Pmyo-2::mCherry]*	This study
AGP105a/b	*glp-1(e2141)III;glmEx20[Punc-119::nhr-49::GFP + Pmyo-2::mCherry]*	This study
nhr-49;glp-1 **Mutant Background**		
AGP34a	*nhr-49(nr2041)I;glp-1(e2141ts)III;glmEx5 [Pnhr-49::nhr-49::GFP + Pmyo-2::mCherry]*	This study
AGP279	*nhr-49(nr2041)I;glp-1(e2141ts)III;glmEx7 [Pmyo-3::nhr-49::GFP + Pmyo-2::mCherry].* Obtained by crossing AGP36 x AGP12a	This study
AGP43	*nhr-49(nr2041)I;glp-1(e2141)III;glmEx10 [Pgly-19::nhr-49::GFP + Pmyo-2::mCherry]*	This study
AGP54a	*nhr-49(nr2041);glp-1(e2141);glmEx11 [Pcol-12::nhr-49::GFP + Pmyo-2::mCherry]*	This study
AGP70a/b	*nhr-49(nr2041);glp-1(e2141);glmEx13 [Prgef-1::nhr-49::GFP + Pmyo-2::mCherry]*	This study
AGP115a/b	*nhr-49(nr2041);glp-1(e2141);glmEx29 [Punc-119::nhr-49::GFP + Pmyo-2::mCherry]*	This study

**Tissue-specific *nhr-49(et7) gof* Expressing Strains**		
AGP311	*glm21 [Pcol-12::nhr-49(et7)::gfp + Pmyo-2::mCherry]*	This study
AGP312	*glm22 [Punc-119::nhr-49(et7)::gfp + Pmyo-2::mCherry]*	This study
AGP313	*nhr-49(nr2041)I; glm22 [Punc-119::nhr-49(et7)::gfp + Pmyo-2::mCherry]*	This study

Table S11: Primers for cloning and qPCR experiments used in this study.

#	Transgene	Description	Forward Primer	Reverse Primer
1	*Pnhr-49::nhr-49::GFP*	Endogenous promoter	GCTAGCCTGCAGGGGACCAGAAAGAGCAAGAGCC	TAAGCAGTCGACATCGTGAGAACGGAATGAGTTG
2	*Pcol-12::nhr-49::GFP*	Hypodermis Specific	GCTAGCCTGCAGGTCAGTATTTGCTATTGAC	TAAGCAGTCGACTTTTCTAAAAAGTAATCAAATC
3	*Pgly-19::nhr-49::GFP*	Intestine Specific	GCTAGCCTGCAGGCGACCGCCGATTGATTGGGG	TAAGCAGTCGACCAGAATTGAGAGTTCTCAATG
4	*Pmyo-3::nhr-49::GFP*	Muscle Specific	GCTAGCCTGCAGGAGTGATTATAGTCTCTGTTT	TAAGCAGTCGACCATTTCTAGATGGATCTAGT
5	*Punc-119::nhr-49::GFP*	Neuron Specific	GCTAGCCTGCAGGAGTAAAAGAAGTAGAATTTTATAG	TAAGCAGTCGACATATGCTGTTGTAGCTGAAAATTTTG
6	*prgef-1::nhr-49::GFP*	Neuron Specific	GCTAGCCTGCAGGCGCAACATTGAATTCCGACCAAGAGC	TAAGCAGTCGACCATCGTCGTCGTCGTCGATGCCGTCTTCACGA
7	*Pcol-12::nhr-49(et7)::GFP*	Gain of Function	CTTGCAGCTCtATTGGCAATTC	AGTTGCTGAGAGCATTCC
8	*Punc-119::nhr-49(et7)::GFP*			
	**Gene**	**Description**	**Forward Primer**	**Reverse Primer**
9	*nhr-49*	qPCR Primer	TTGGCAGAGGTGGATTCTC	CTGTAAAGAGACCGGAGCC
10	*rpl-32*	qPCR Primer (Houseke	GATTCCCTTGCGGCTCTT	GATTCCCTTGCGGCTCTT
